# Supplementary material for: Symptom networks of common mental disorders in public versus private healthcare settings in India
Source: Glob Ment Health (Camb). 2025 Feb 17;12:e30. doi: 10.1017/gmh.2025.16 (PMC11894409; doi:10.1017/gmh.2025.16)

SUPPLEMENTARY MATERIAL

NETWORK NODES (SYMPTOM SUBSCALE DESCRIPTIONS)

A total of fourteen symptom scores were entered in the networks as nodes. All twelve subscale scores were used as assessed by the CIS-R. The symptom subscale scores range from 0 to 4, except for fatigue and the depressive ideas, which ranges from 0 to 5. When binarized, scores that are equal to or higher than 2 were coded as "1" as in the original CIS, these scores were thought to indicate clinical severity of a symptom, while scores that are less than 2 are thought to indicate absence of symptom, habitual traits, or borderline subclinical levels.

- Somatic symptoms, consisting of items relating to the respondent's experience of any ache, pain or discomfort (e.g., "Have you had any sort of ache or pain in the last month"?).
- Fatigue, including items related to feeling tired or lacking in energy (e.g., "Have you felt tired/lacking energy for more than 3 hours in total on any day or most of the day in the past seven days?").
- Concentration and forgetfulness, consisting of items relating to experience of problems with memory and/or concentration (e.g., "Have you noticed any problem with forgetting things in the past month?").
- Sleep problems, assessing problems with trying to get to sleep, waking up too early, or sleeping more than is usual (e.g., "In the past seven days, on how many nights did you spend three or more hours trying to get to sleep?")
- Irritability, relates to feelings of irritability, being short-tempered or angry (e.g.,"Many people become irritable or short tempered at times, though they may not show it. Have you felt irritable or short tempered with those around you in the past month?")
- Worry about physical health, includes items asking about the extent of health-related worries (e.g., "In your opinion, have you been worrying too much in view of your actual health?").
- Depressed mood, relating to respondents' feelings of sadness, misery or depressed or unable to enjoy or take an interest in things as much as usual (e.g.,"Almost everyone becomes sad, miserable, or depressed at times. Have you had a spell of feeling sad, miserable or depressed in the past month?").
- Depressive ideas, captures diurnal variation, restlessness, psychomotor agitation, feeling guilty, blaming him/herself when things went wrong, worthlessness, hopelessness, and suicidal ideas (e.g., "In the past seven days, have you thought of a way in which you might kill yourself?").
- Worry, captures worries about things and circumstances, other than the physical health (e.g., "On how many of the past seven days have you been worrying about things other than your physical health?").
- Anxiety, includes items asking about anxious feelings, nervousness or tension (e.g., "When anxious, nervous or tense, had one or more of following symptoms: heart racing or pounding, hands sweating or shaking, feeling dizzy, difficulty getting breath, butterflies in stomach, dry mouth, nausea or feeling as though he/she wanted to vomit?").
- Phobias, assesses experience of phobia or avoidance related to specific situations (e.g., "Can you tell me which of the situations or things made you the most anxious/nervous in the past month: crowds or public places, enclosed spaces, social situations, sight or blood or injury, specific single cause, others?").
- Panic, includes items asking about feelings of panic, or of collapsing and losing control (e.g., "Thinking about the last month, did your anxiety or tension ever get so bad that you got in a panic, for instance make you feel that you might collapse or lose control unless you did something about it?").

Two additional variables were computed manually and included in the network analysis. The appetite and functioning variables. The appetite and weight change had three levels, (1 = “no change”, 2 = “appetite increase/decrease”, 3 = “change in appetite and weight”). When treated binary, any change in appetite was coded as “1”. The overall effects variable had four levels (1= “no change in functioning”, 2 = “feelings made it difficult even though got everything done, 3 = “impacted functioning once”, 4 = “impacted functioning more than once”). When treated binary, impacted functioning once and impacted functioning more than once were coded as “1”.

- Appetite and weight change, capturing any marked loss or gain of weight and increased or decreased appetite (e.g., “Have you noticed a marked increase in your appetite in the past month?”).
- Overall effects (i.e., functional impairment), assesses whether or not and the extent to which the symptoms have affected the participant (e.g., “Has the way you have been feeling ever actually stopped you from getting on with things you used to do or would like to do?”).

Three other binary variables will be computed to be used in the proposed study:

- CMD case at baseline, computed from the algorithm generating ICD-10 diagnoses from the CIS-R responses (case at baseline versus non-case at baseline)
- CMD case at 6 months, computed from the algorithm generating ICD-10 diagnoses from the CIS-R responses (case at 6 months versus non-case 6 months)
- CMD case at 12 months, computed from the algorithm generating ICD-10 diagnoses from the CIS-R responses (case at 12 months versus non-case 12 months)

For those who met diagnostic criteria for a CMD at baseline, the variable “treatment status” with four mutually exclusive levels will be computed based on the CMD case status at 6 and 12 months:
• Sustained remission refers to those who were non-case across at both 6 and 12 months.
• Relapse at 12 months, refers to those who were non-case at 6 months but were cases at 12 months.
• Delayed remission, refers to those who were cases at 6 months but were no longer cases at 12 months
• Non-remission, refers to those who were cases at both 6 months and 12 months

GGM RESULTS (AIM 1)

Figure 1. The GGM network, average correlation of the bootstrapped indices with the original sample (network stability)

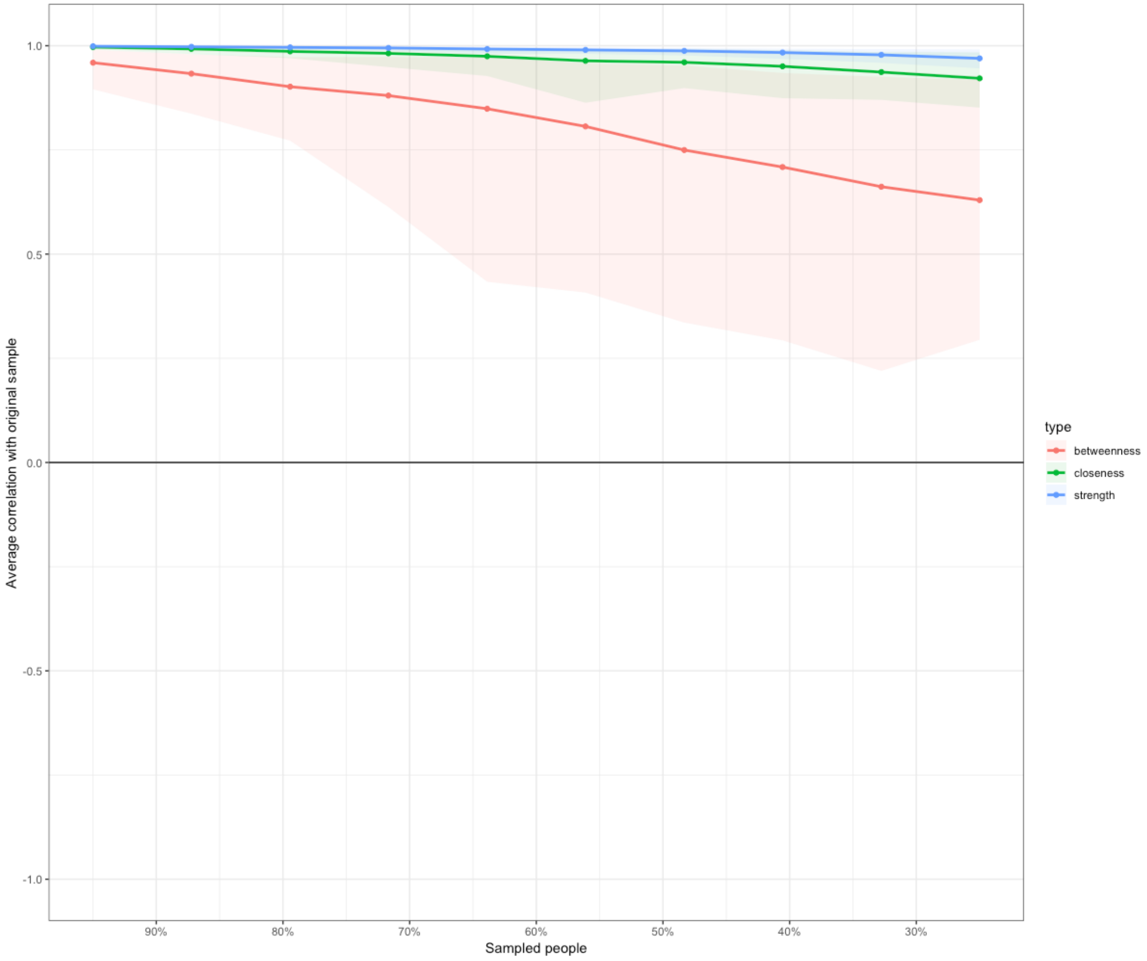

Figure 2. The GGM network, bootstrapped edge-weights and the confidence intervals (edge-weight accuracy)

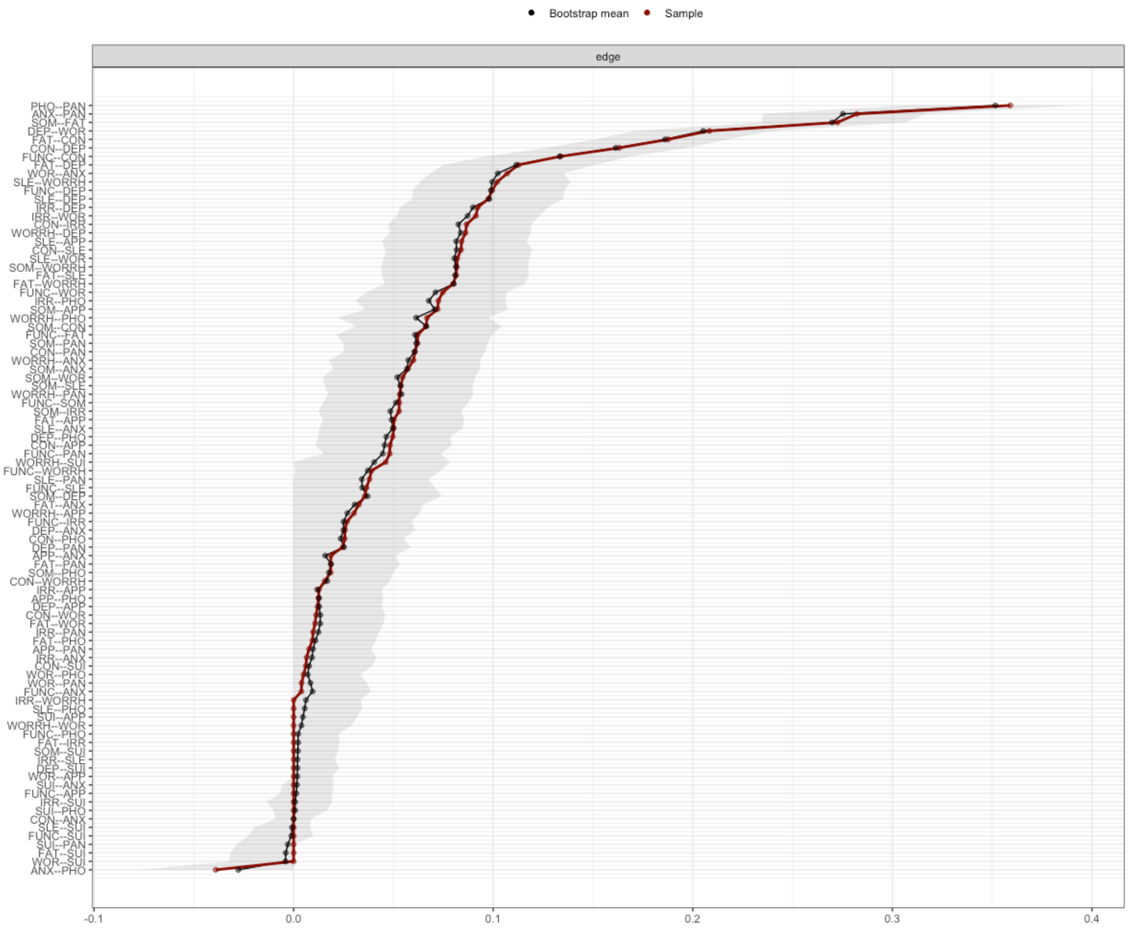

Figure 3. The GGM network, bootstrapped edge-weights and the % of times an edge was estimated as zero (edge-weight stability)

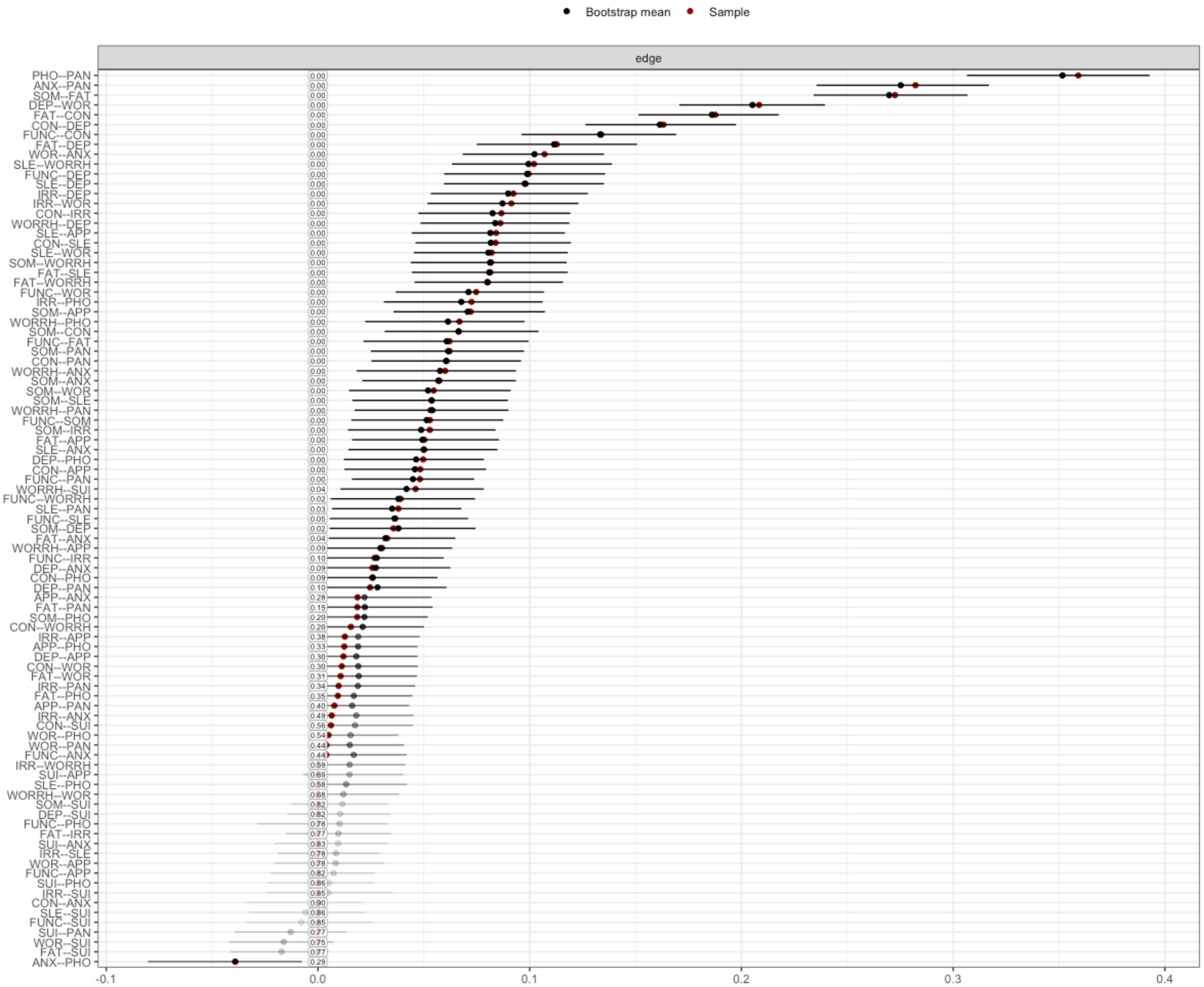

Figure 4. The GGM network, bootstrapped edge-weight differences (black boxes indicate significant difference between the corresponding edge-weights)

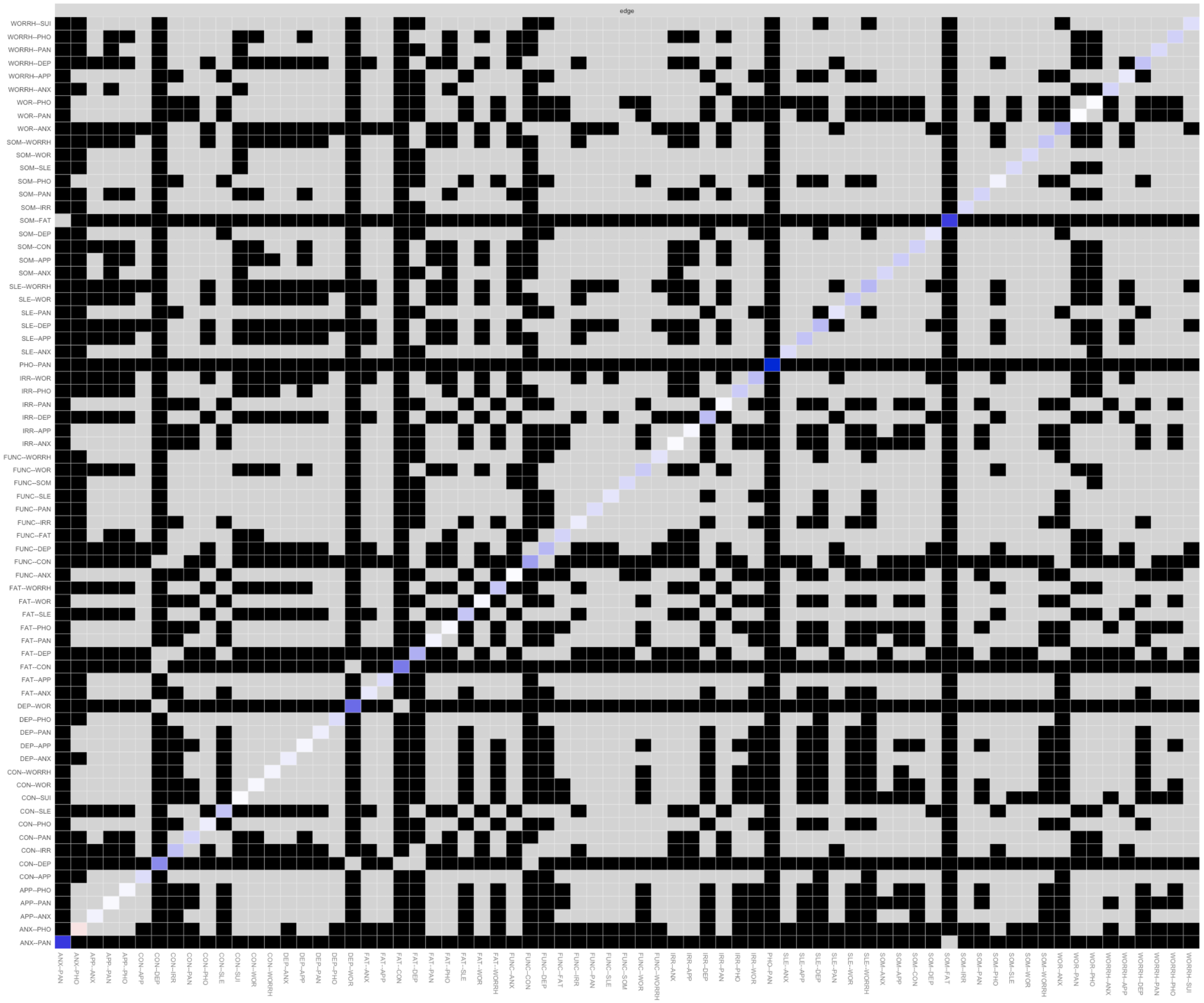

Figure 5. The GGM network, bootstrapped differences of centrality scores (black boxes indicate significant difference)

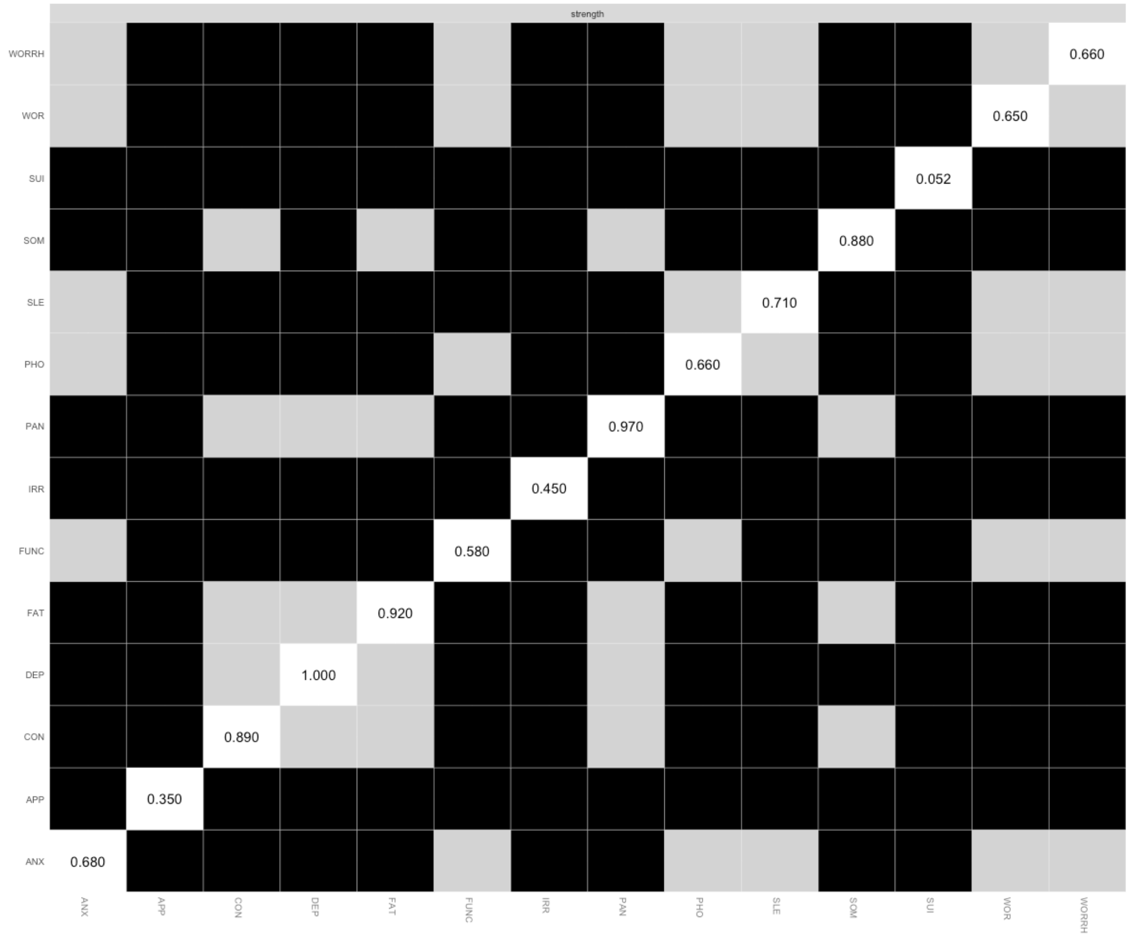

Table 1. The centrality scores centrality indices of the GGM network

Node	Centrality
Depressed mood	1
Panic	0.97
Fatigue	0.92
Concentration problems	0.89
Somatic symptoms	0.88
Sleep	0.71
Anxiety	0.68
Worry about health	0.66
Phobias	0.66
Worry	0.65
Functional impairment	0.58
Irritability	0.45
Appetite loss/gain	0.35
Depressive ideas (including suicidality)	0.052

Note: Centrality scores are z-standardized

GGM RESULTS ACROSS PRIVATE AND PUBLIC HEALTH CARE SETTING (AIM 3)

Figure 6: The GGM network in public settings, average correlation of the bootstrapped indices with the original sample (network stability)

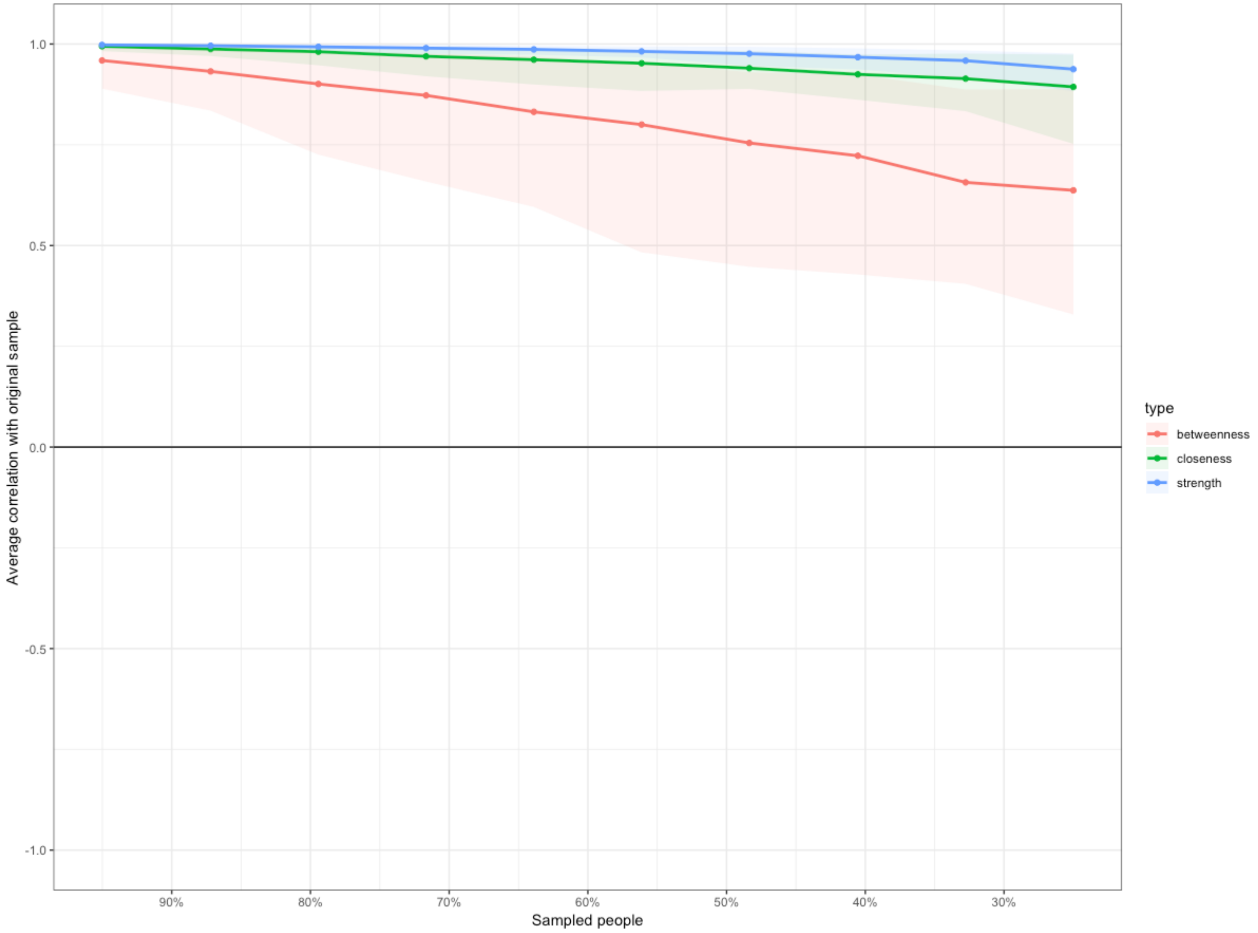

Figure 7. The GGM network in public settings, bootstrapped edge-weights and the % of times an edge was estimated as zero (edge-weight stability

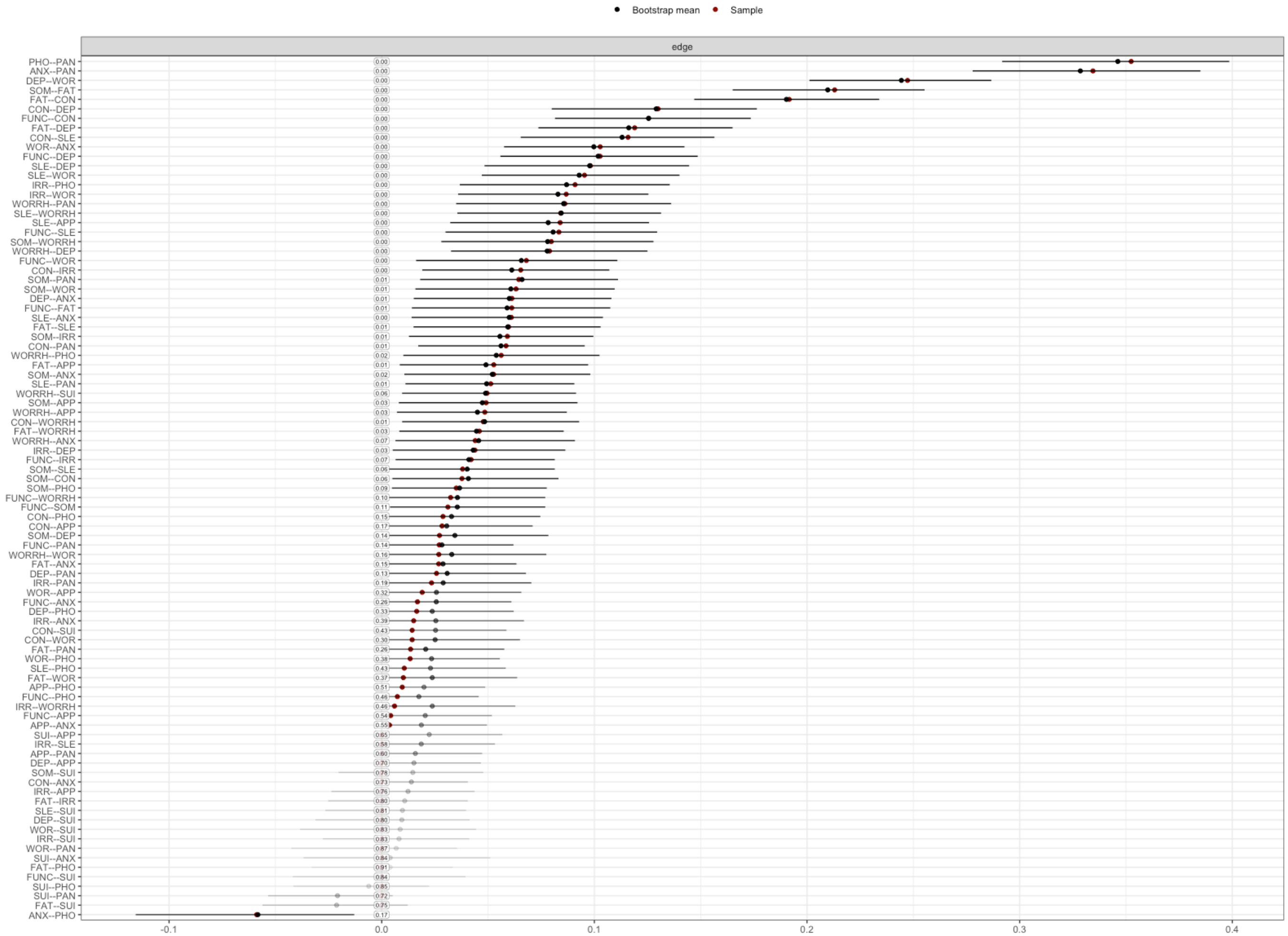

Figure 8. The GGM centrality in public settings

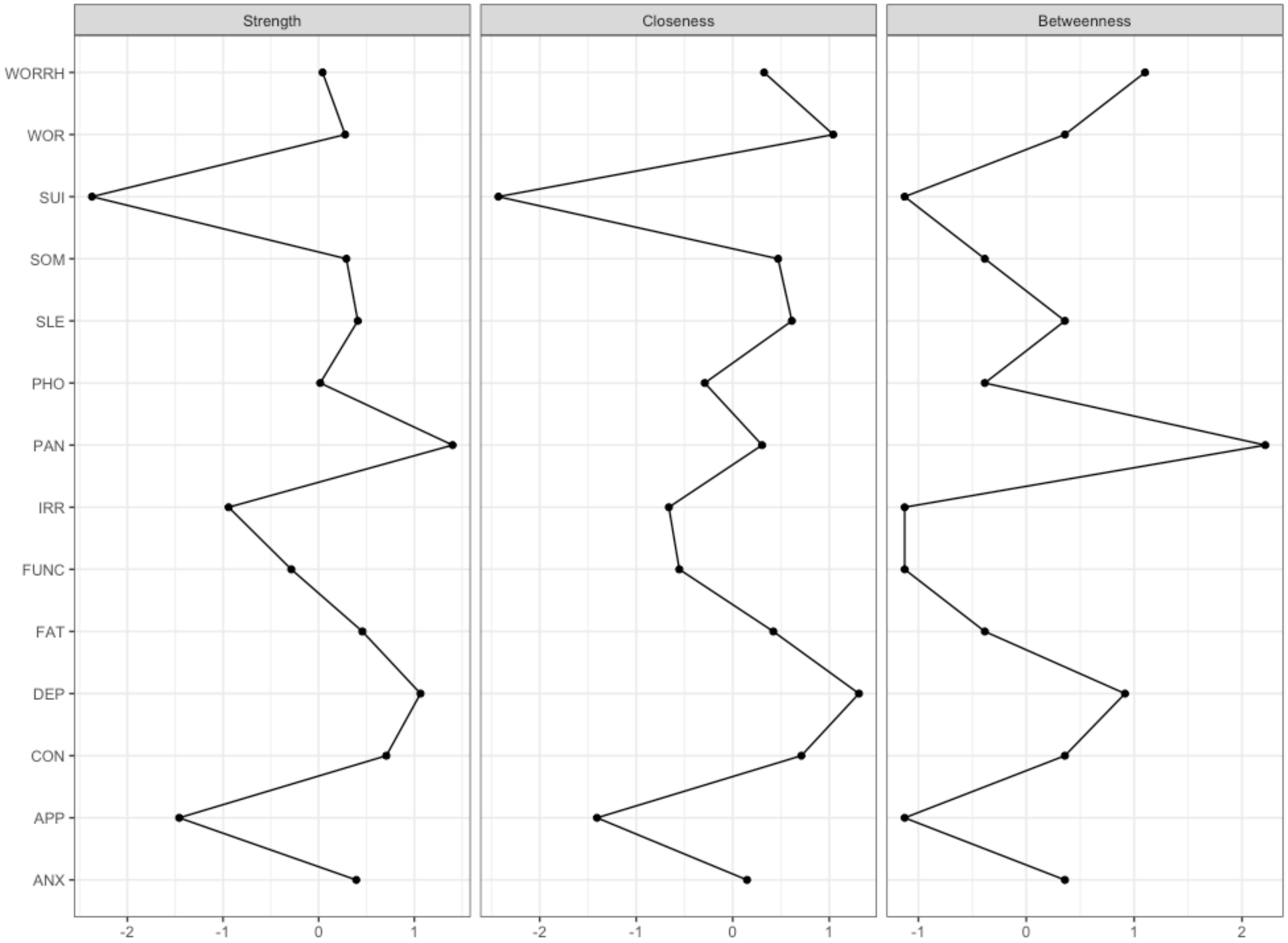

Figure 9. GGM network, public health care, bootstrapped differences of centrality scores (black boxes indicate significant difference)

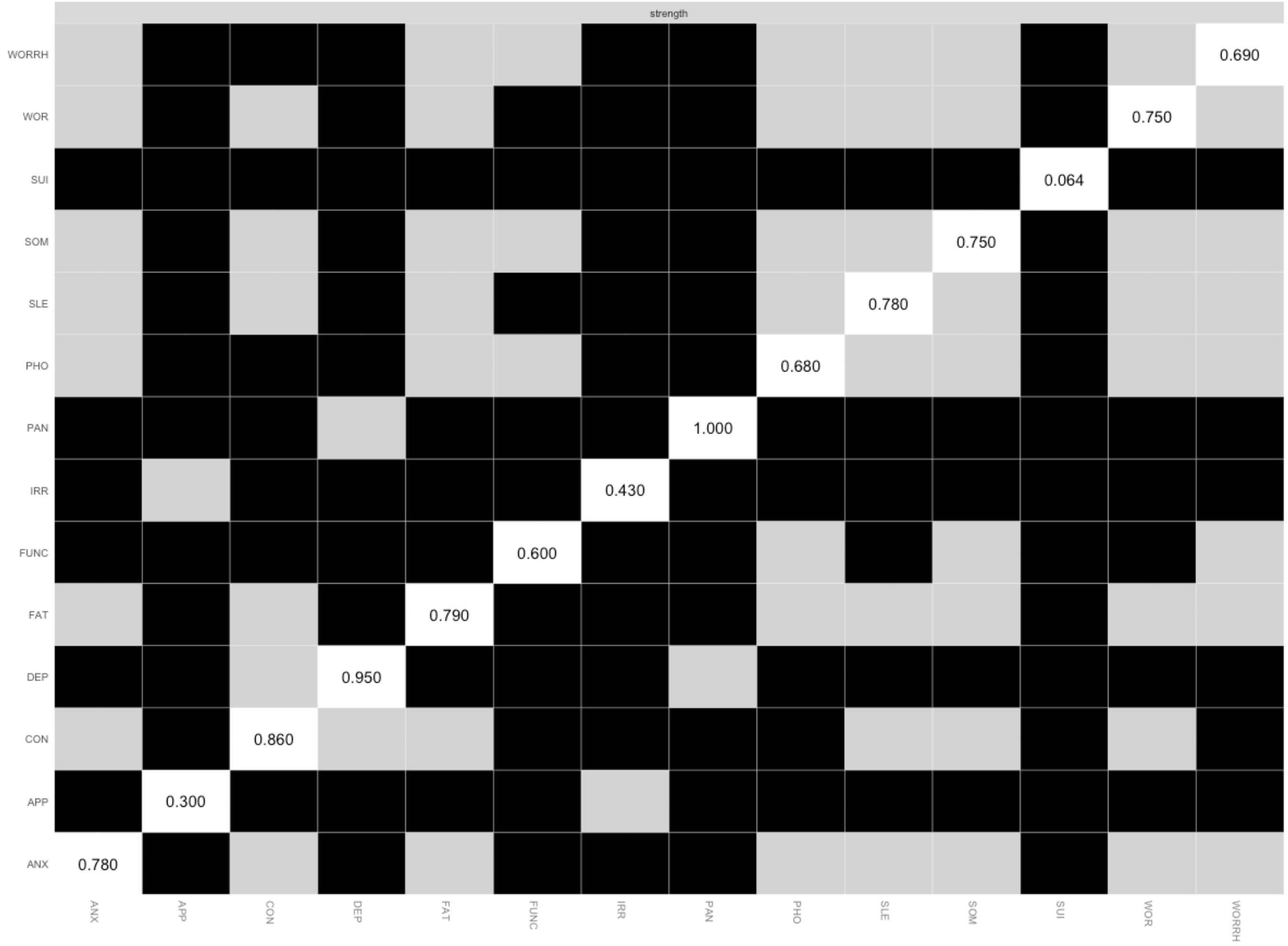

Figure 10. GGM network, public health care, bootstrapped differences of edge-weights (black boxes indicate significant difference)

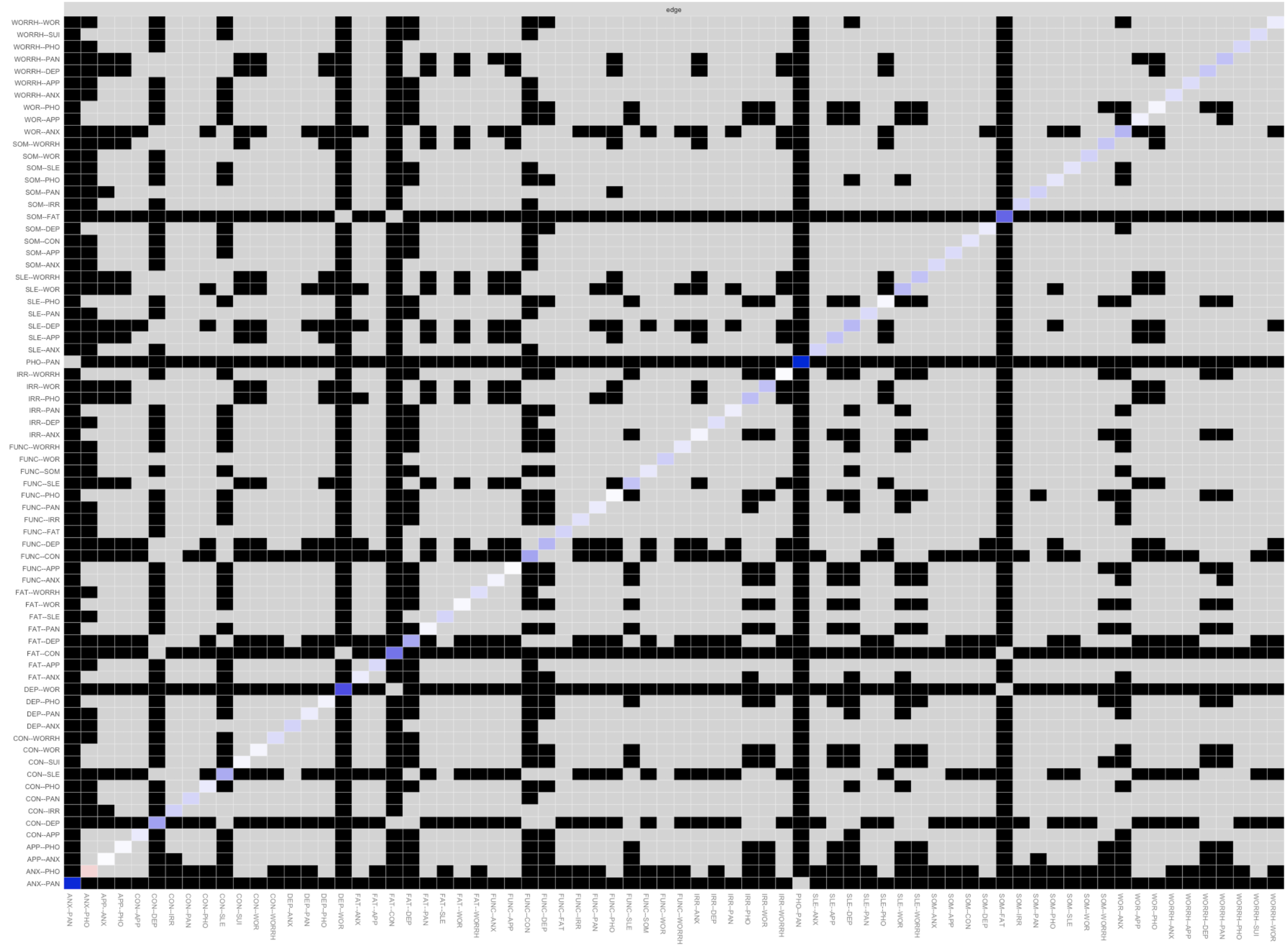

Figure 11. The GGM network in private settings, average correlation of the bootstrapped indices with the original sample (network stability)

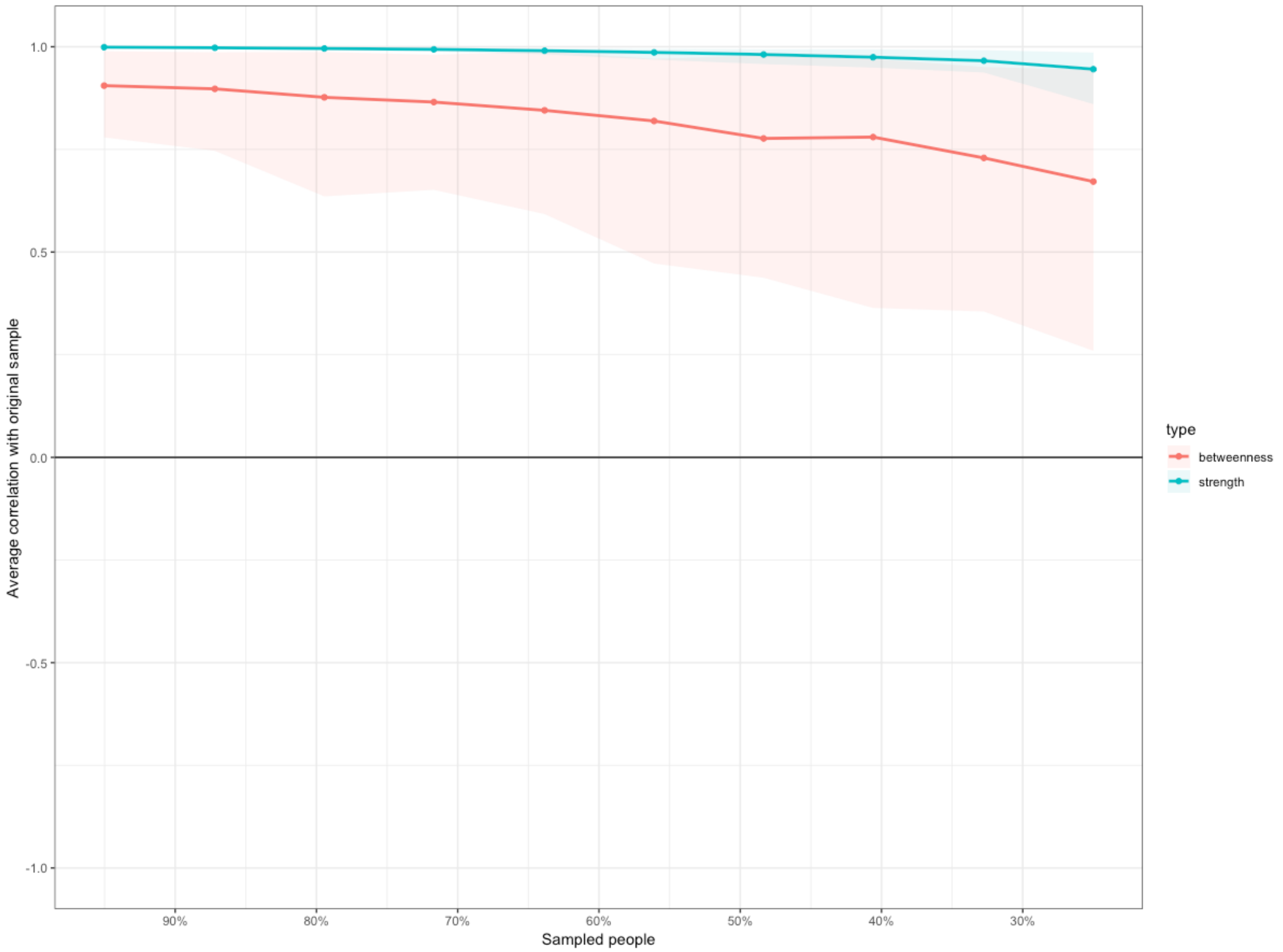

Figure 12. The GGM network in private settings, bootstrapped edge-weights and the % of times an edge was estimated as zero (edge-weight stability)

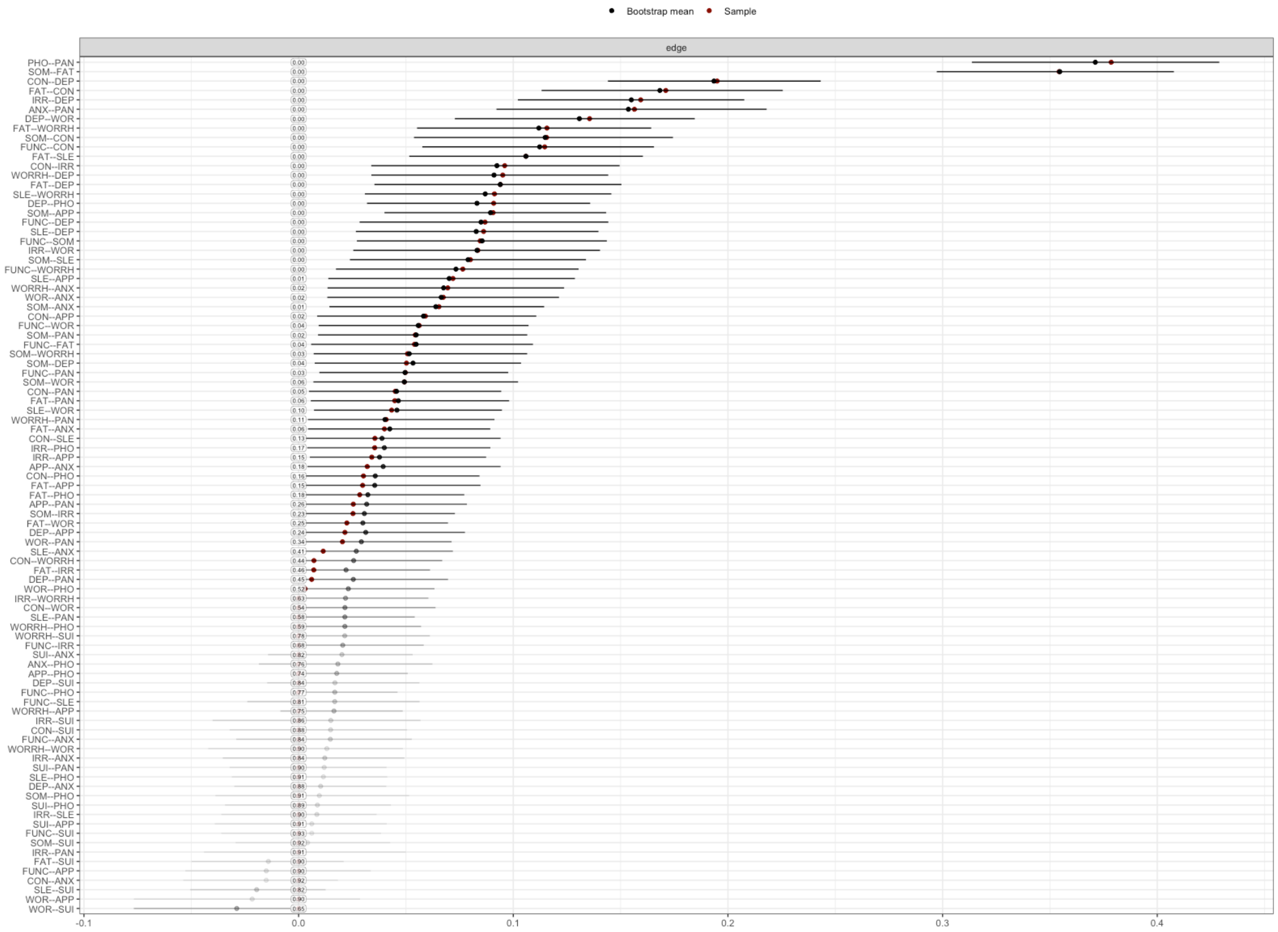

Figure 13. The GGM centrality in public settings

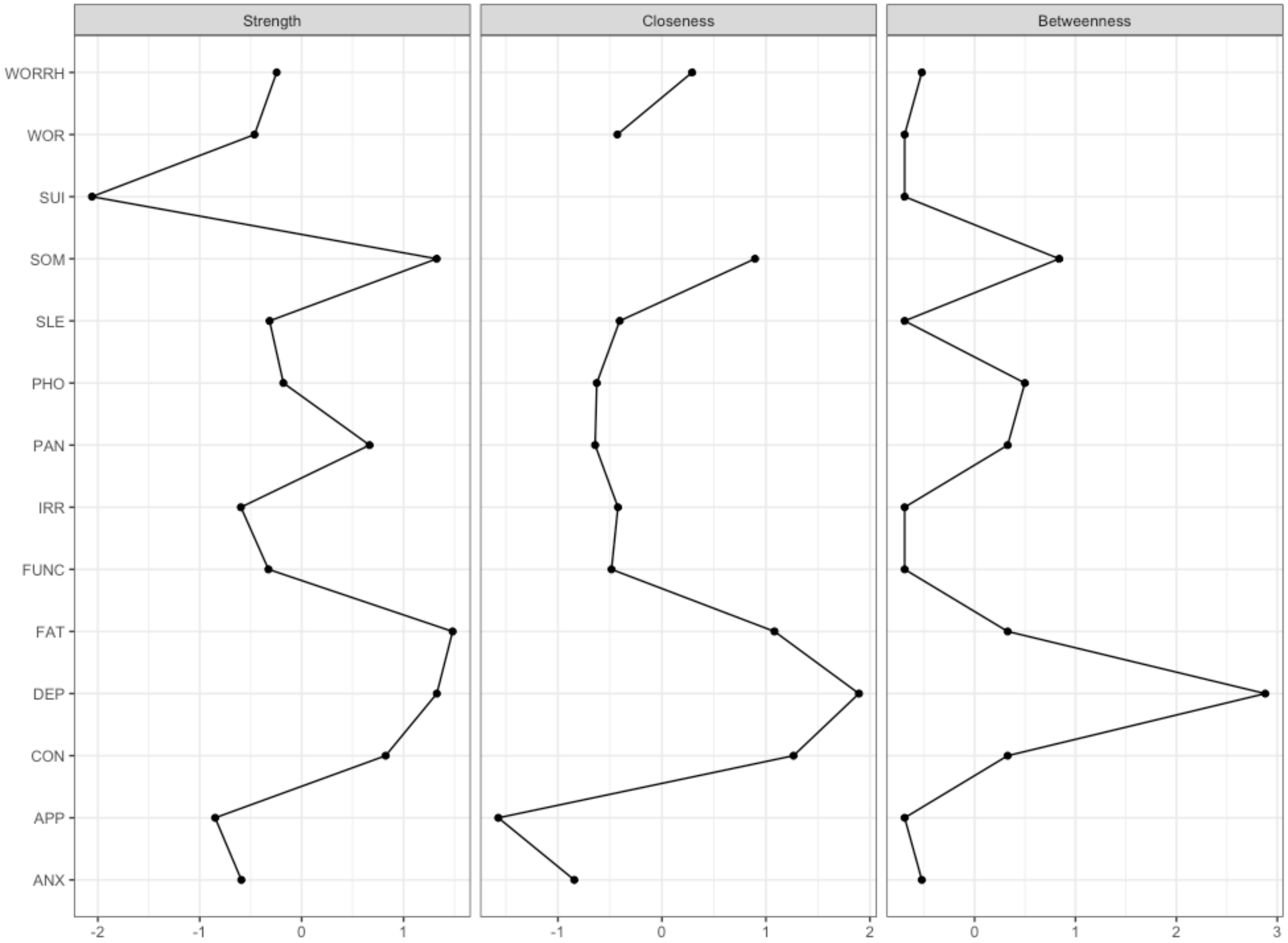

Figure 14. GGM network, private health care, bootstrapped differences of centrality scores (black boxes indicate significant difference)

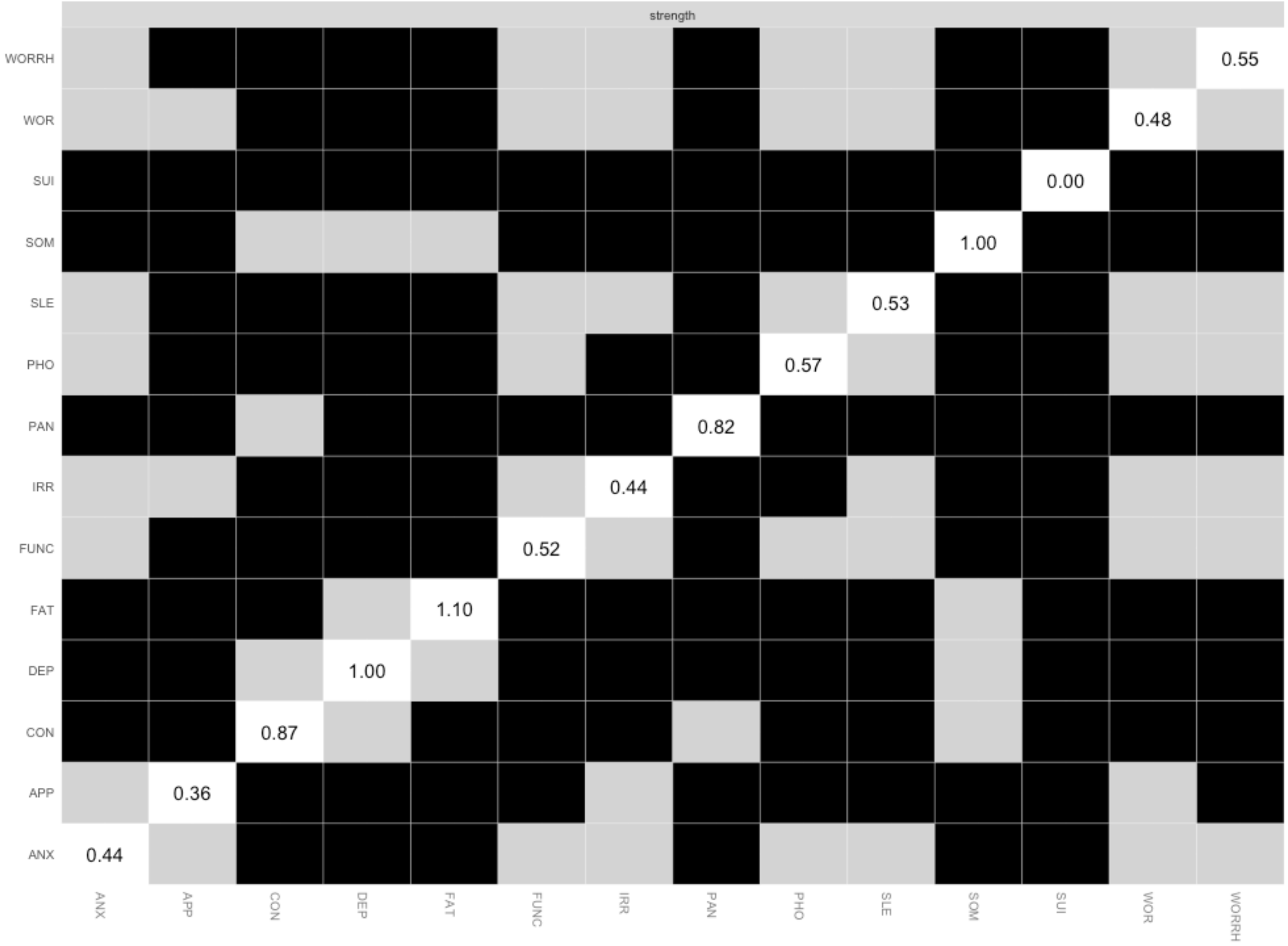

Figure 15. GGM network, private health care, bootstrapped differences of edge-weights (black boxes indicate significant difference)

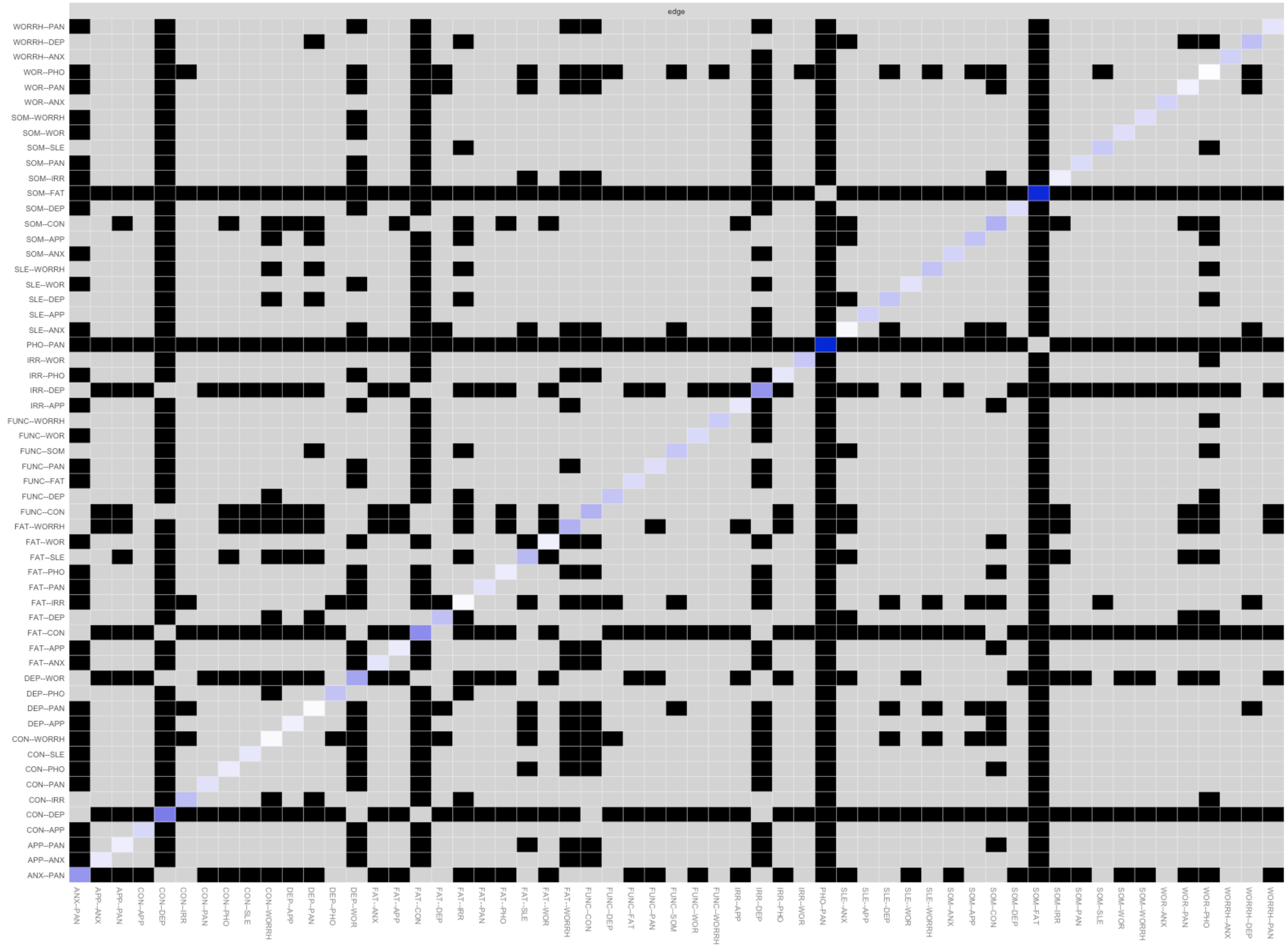

Figure 16. Ising Model

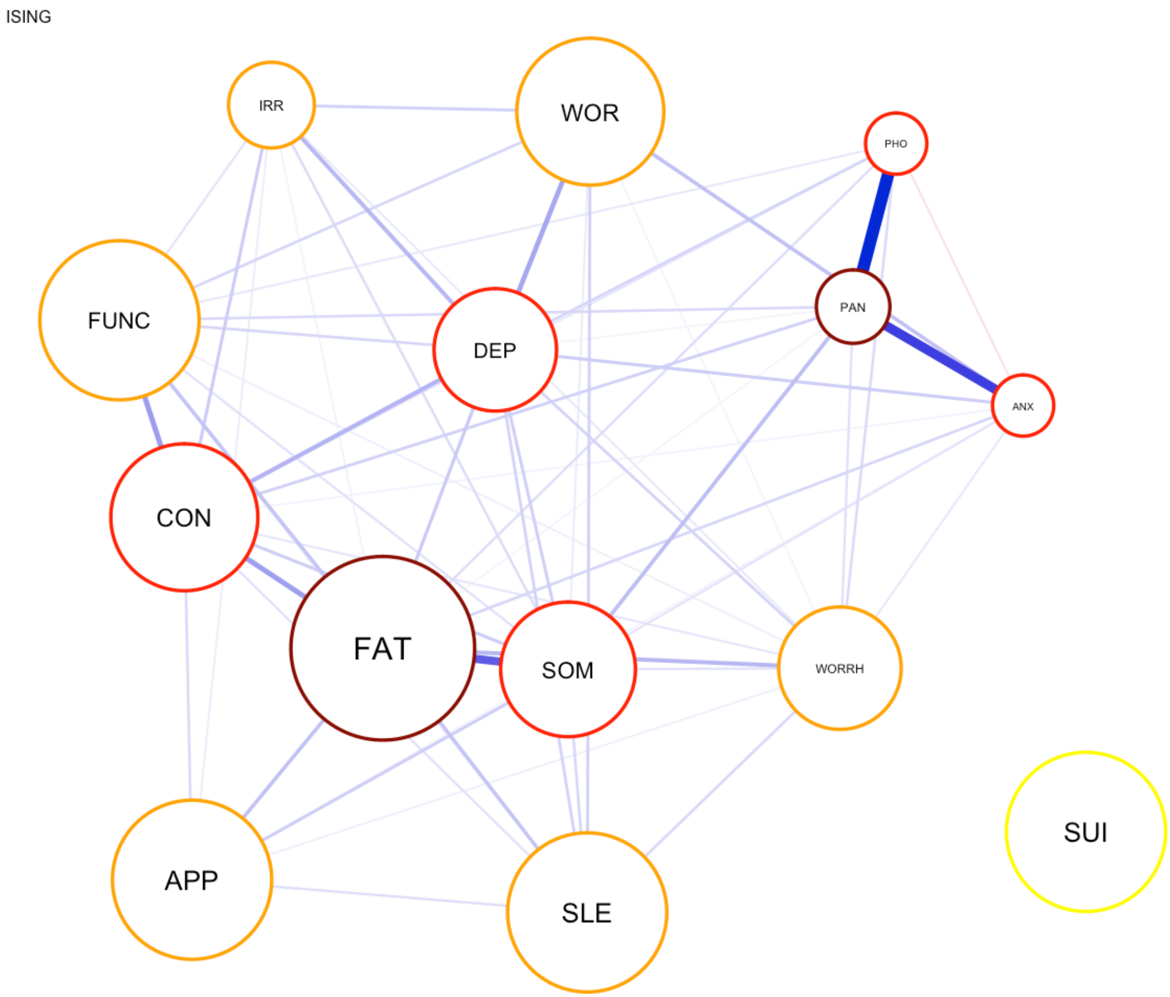

Figure 17. Ising Model, average correlation of the bootstrapped indices with the original sample (network stability)

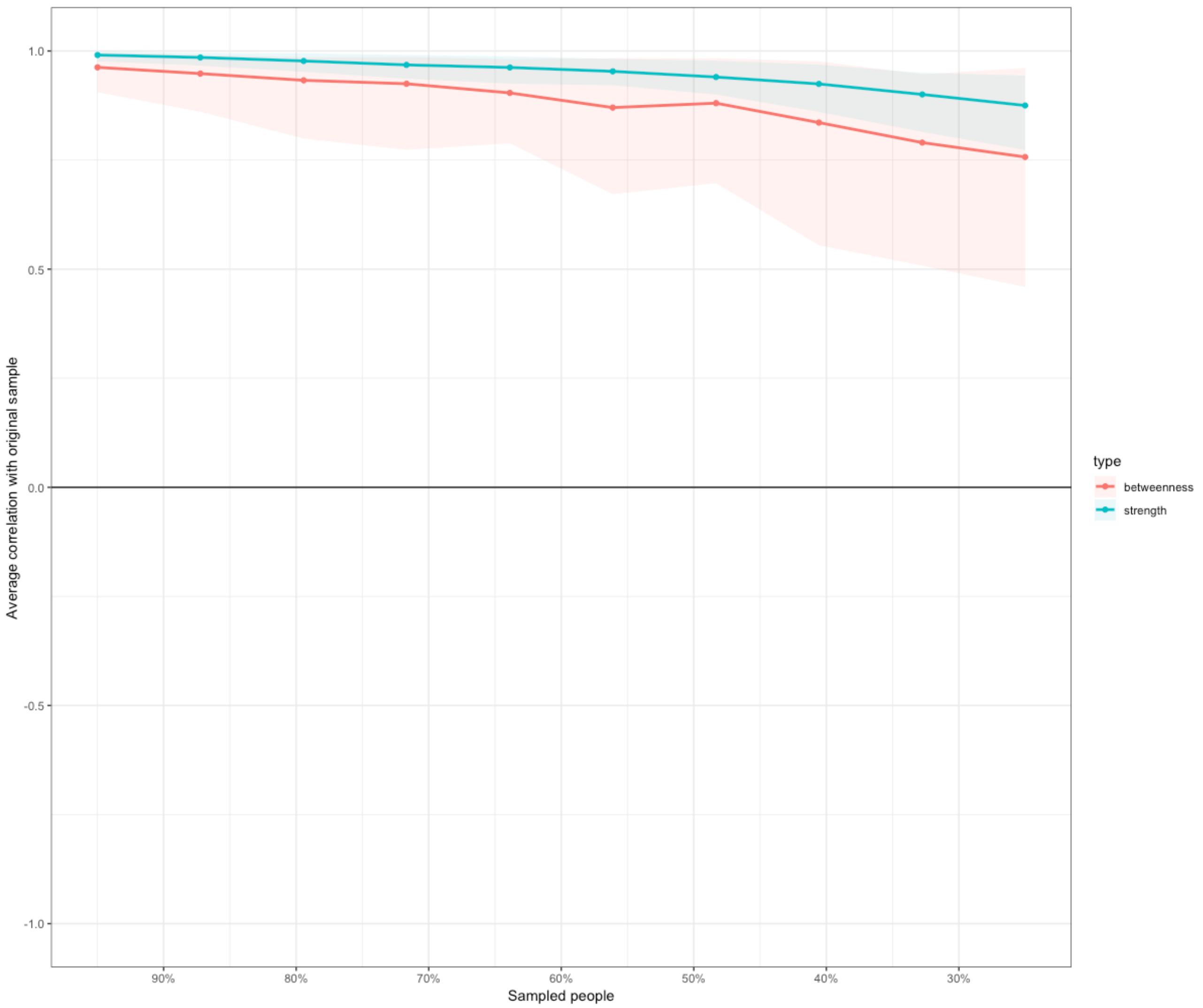

Figure 18. Ising Model, centrality indices

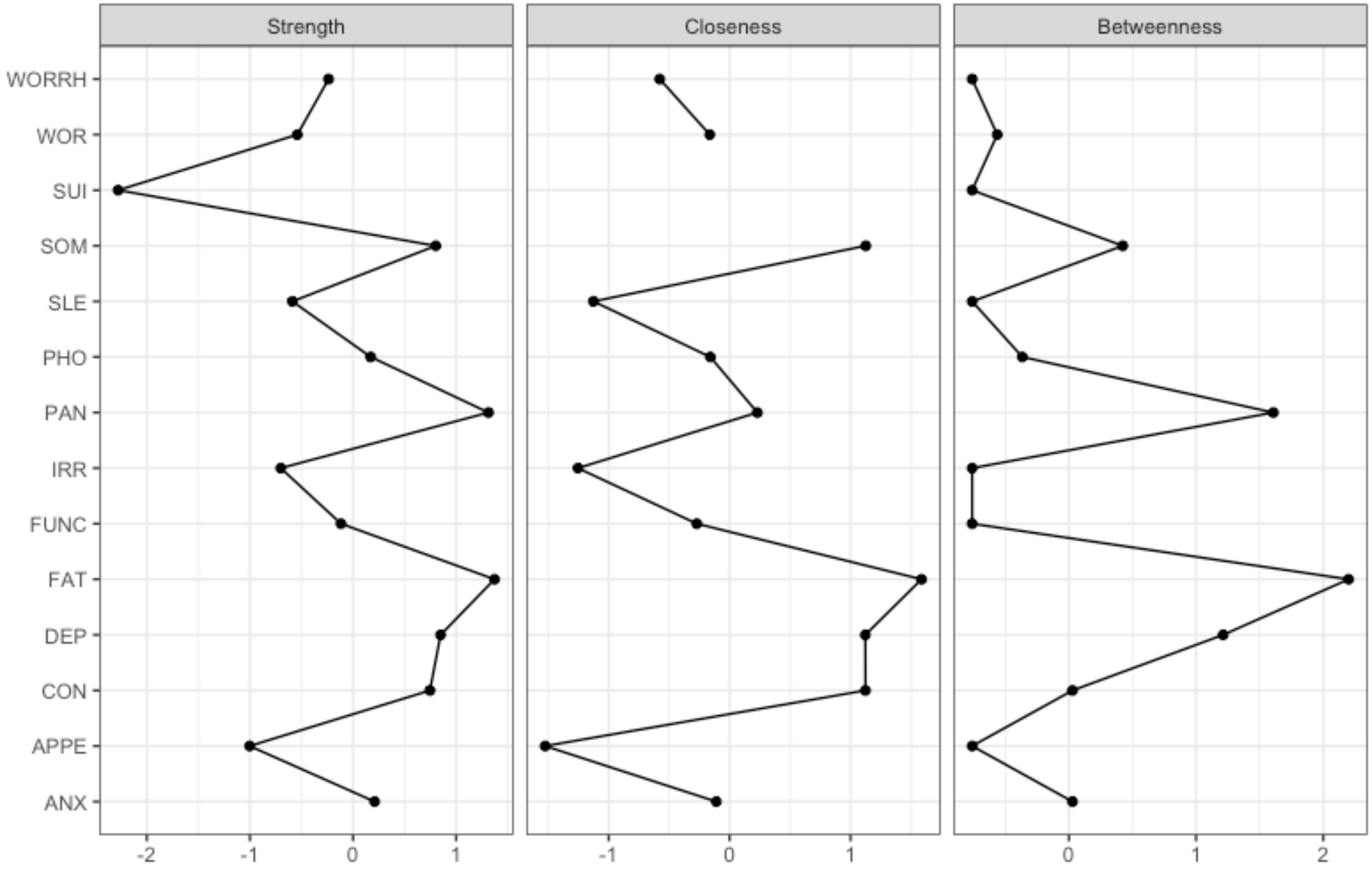

Figure 19. Ising Model, bootstrapped differences of the centrality indices (black boxes indicate significant difference)

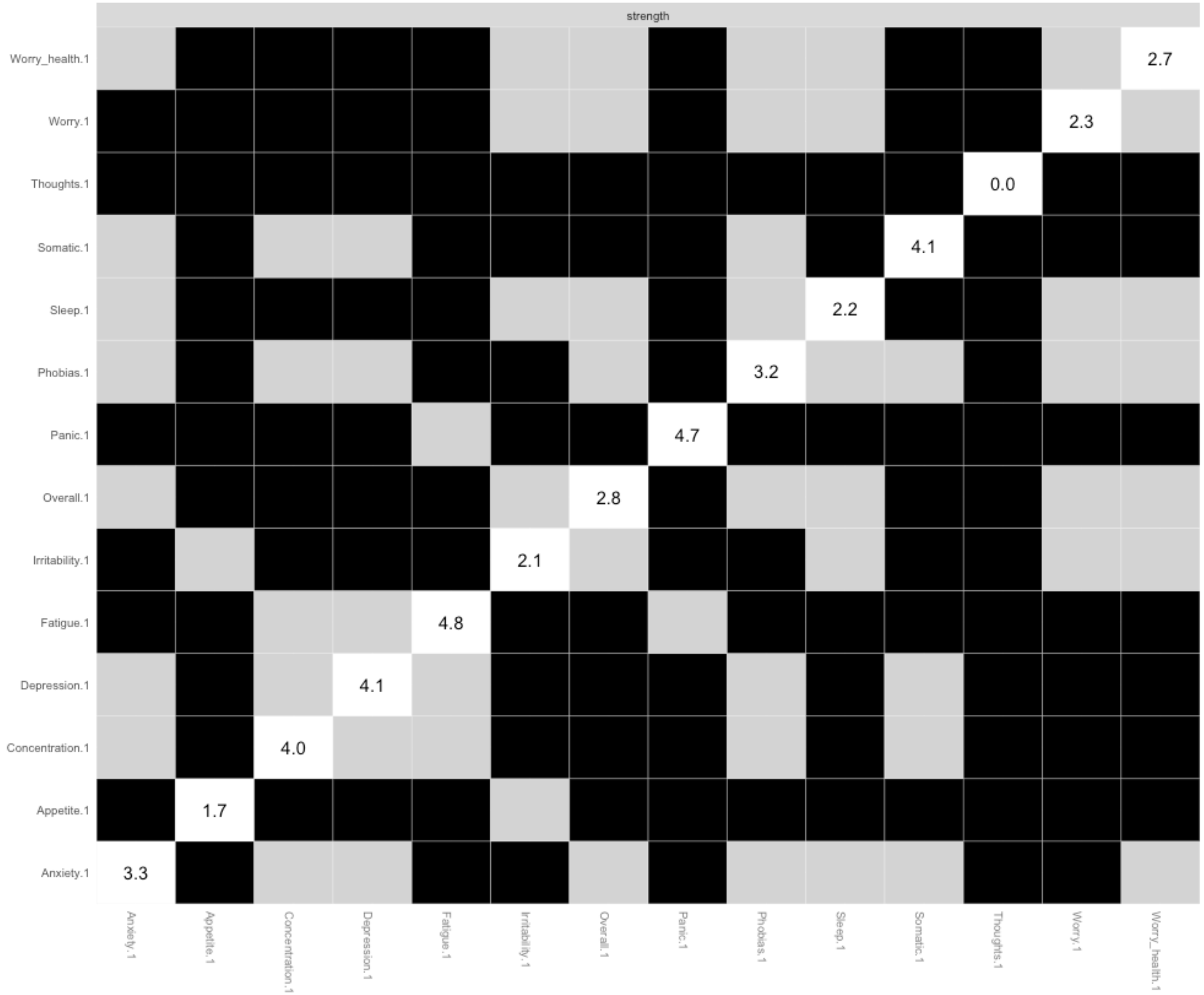

Figure 20. Ising Model, bootstrapped edge-weights, the CIs, and % of times the edges were estimated as zero (edge-weight stability)

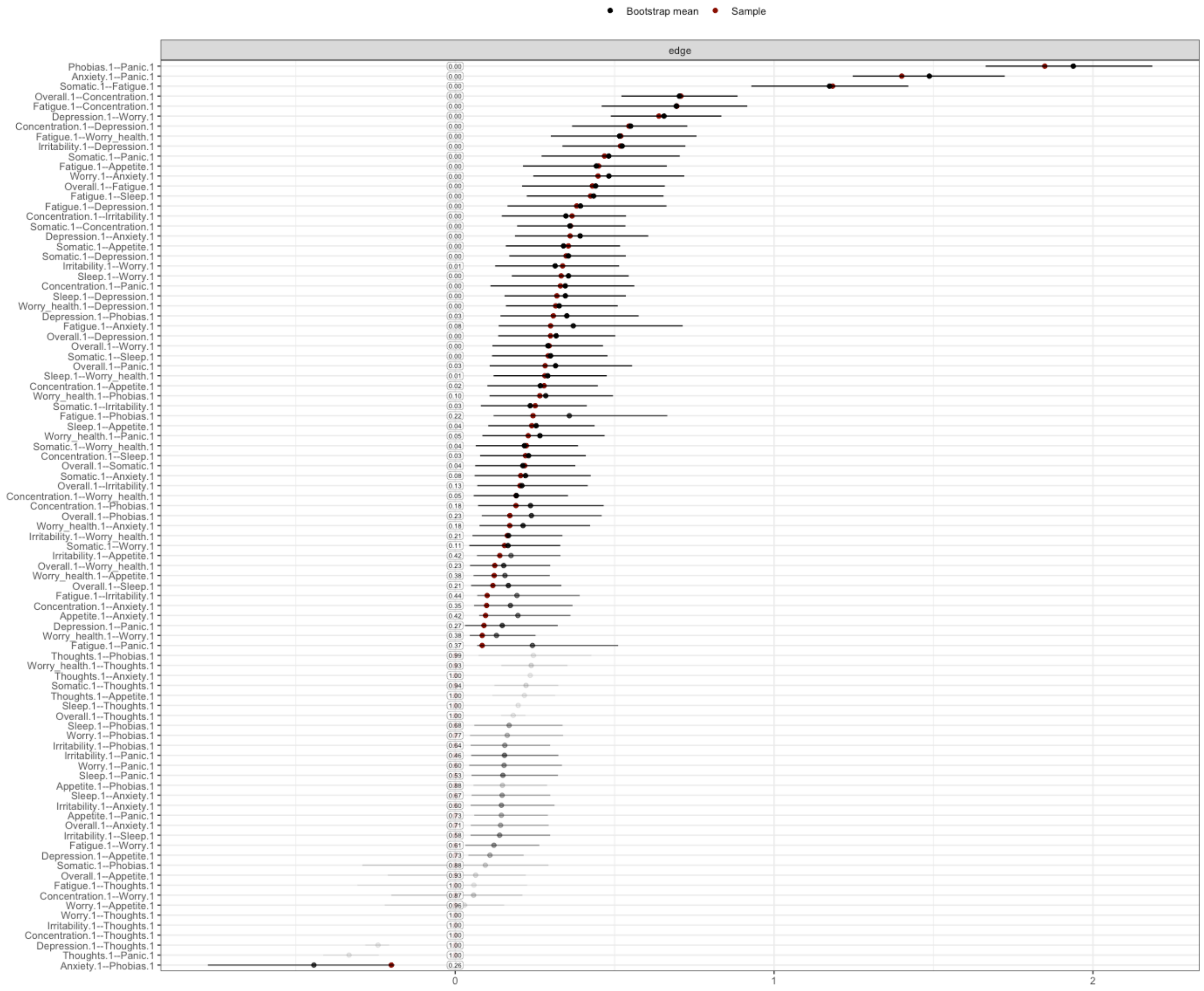

Figure 21. Mixed graph model (MGM) network

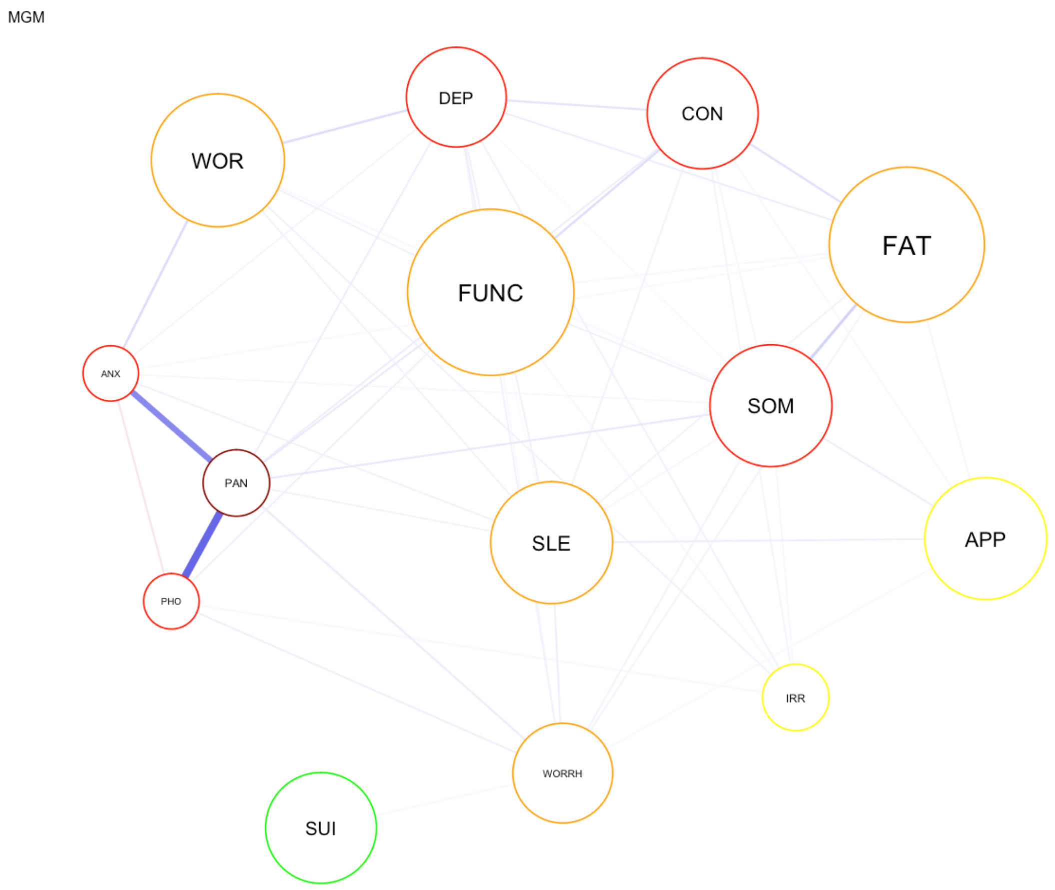

Note 1: The size of the nodes represents their mean value. The colors represent node centrality in following decreasing order; *dark red, red, orange, yellow, green.* APP: Appetite and weight changes, ANX: Anxiety, CON: Concentration, DEP: Depression, FAT: Fatigue, FUNC: Functional impairment, IRR: Irritability, PAN: Panic, PHO: Phobia, SOM: Somatic, SLE: Sleep problems, SUI: Depressive ideas, WOR: Worry, WORRH: Worry about health.

Figure 22. The MGM network, centrality indices of the CMD symptoms

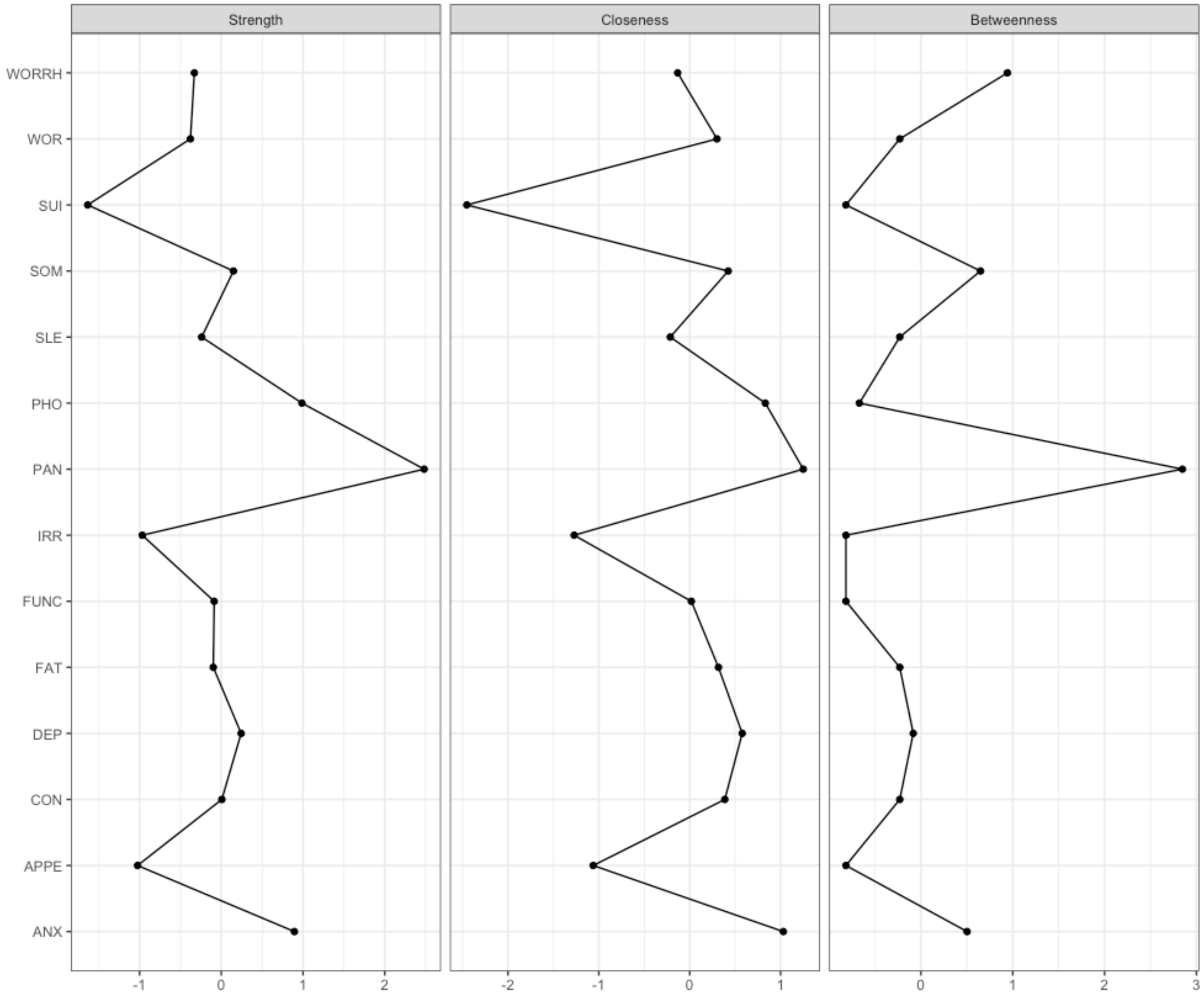

Figure 23. The MGM network, average correlation of the bootstrapped indices with the original sample (network stability)

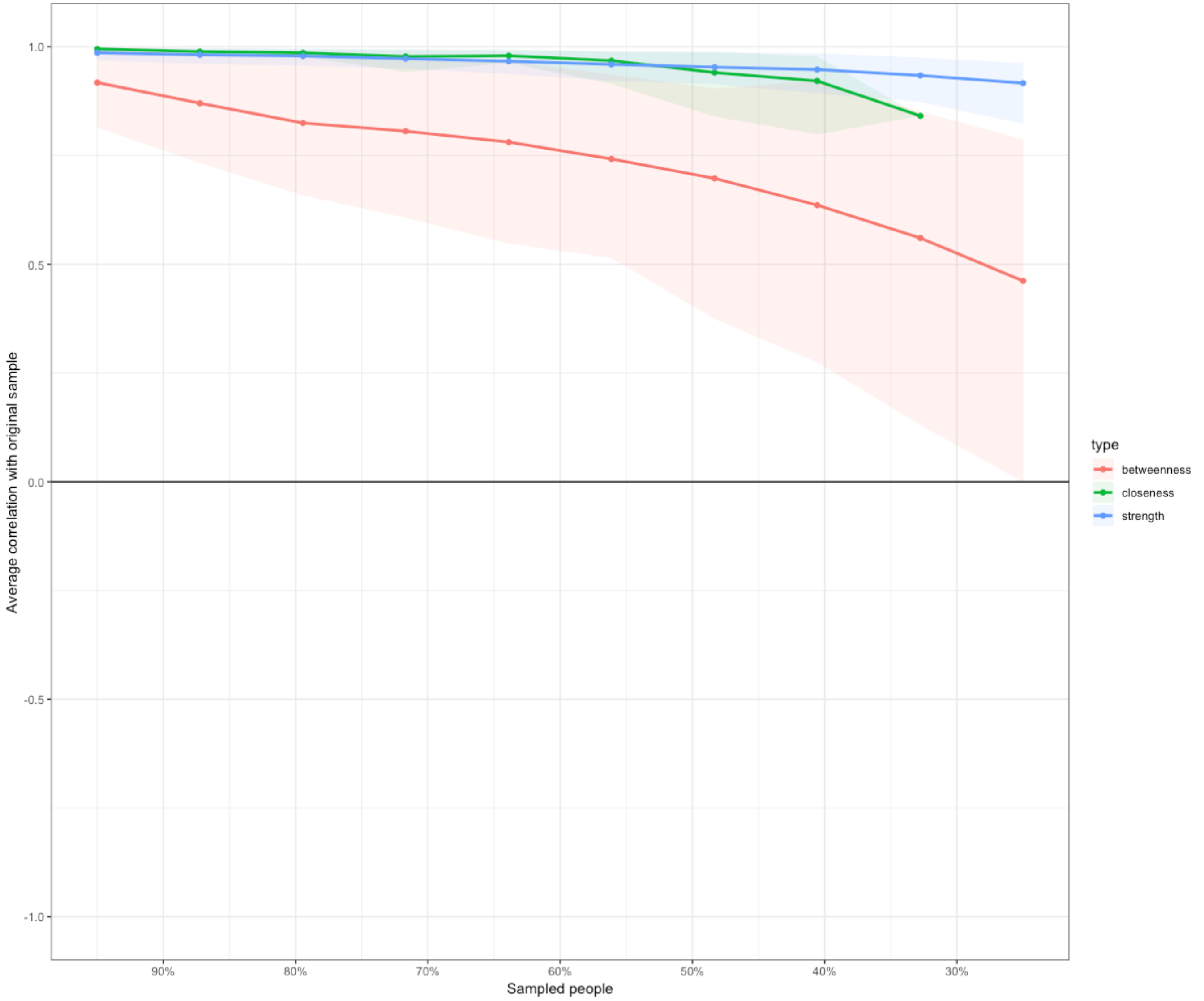

Figure 24. The MGM network, bootstrapped differences of the centrality indices (black boxes indicate significant difference)

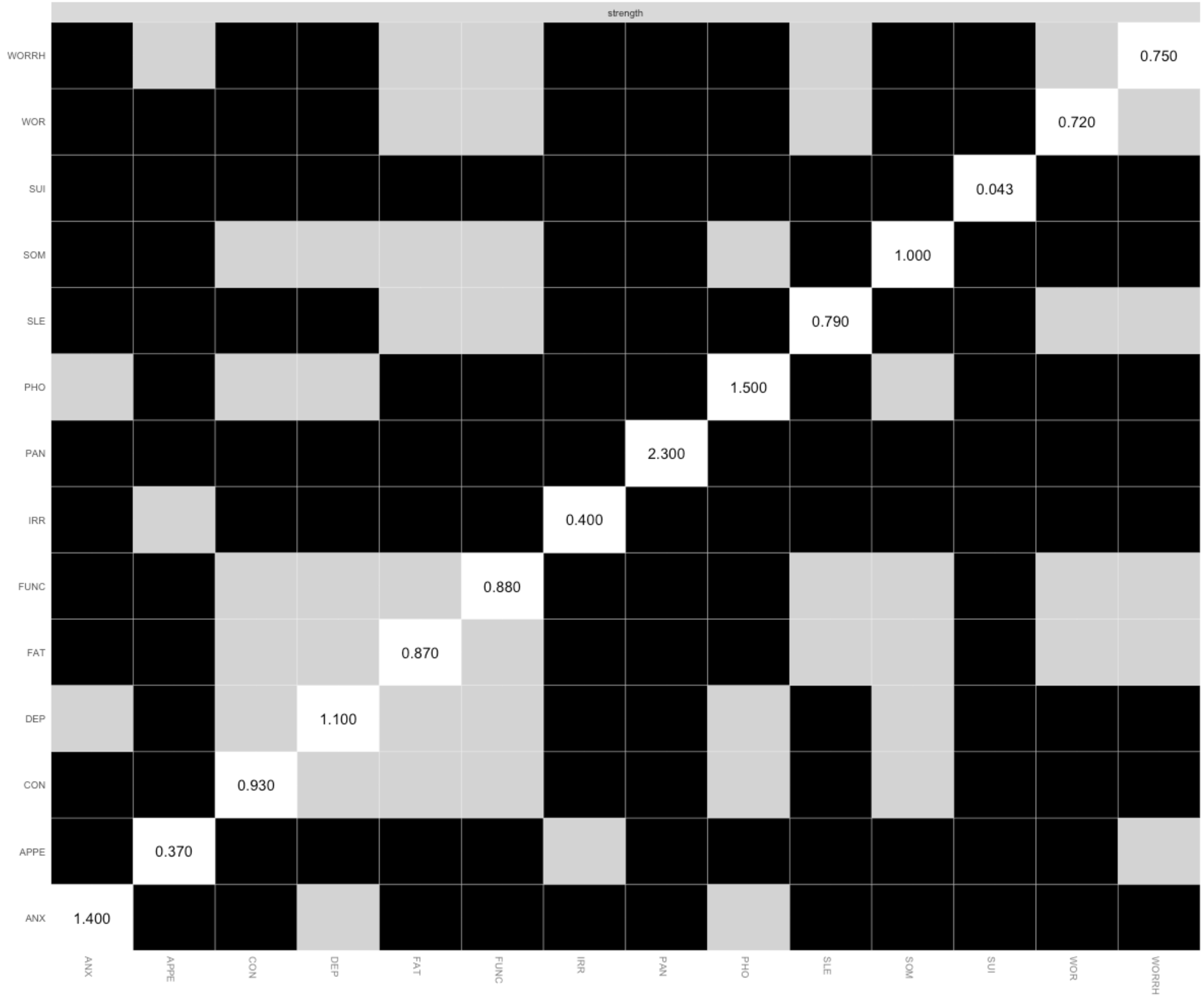

Figure 25. The MGM network, bootstrapped edge-weights and the CIs (edge-weight accuracy)

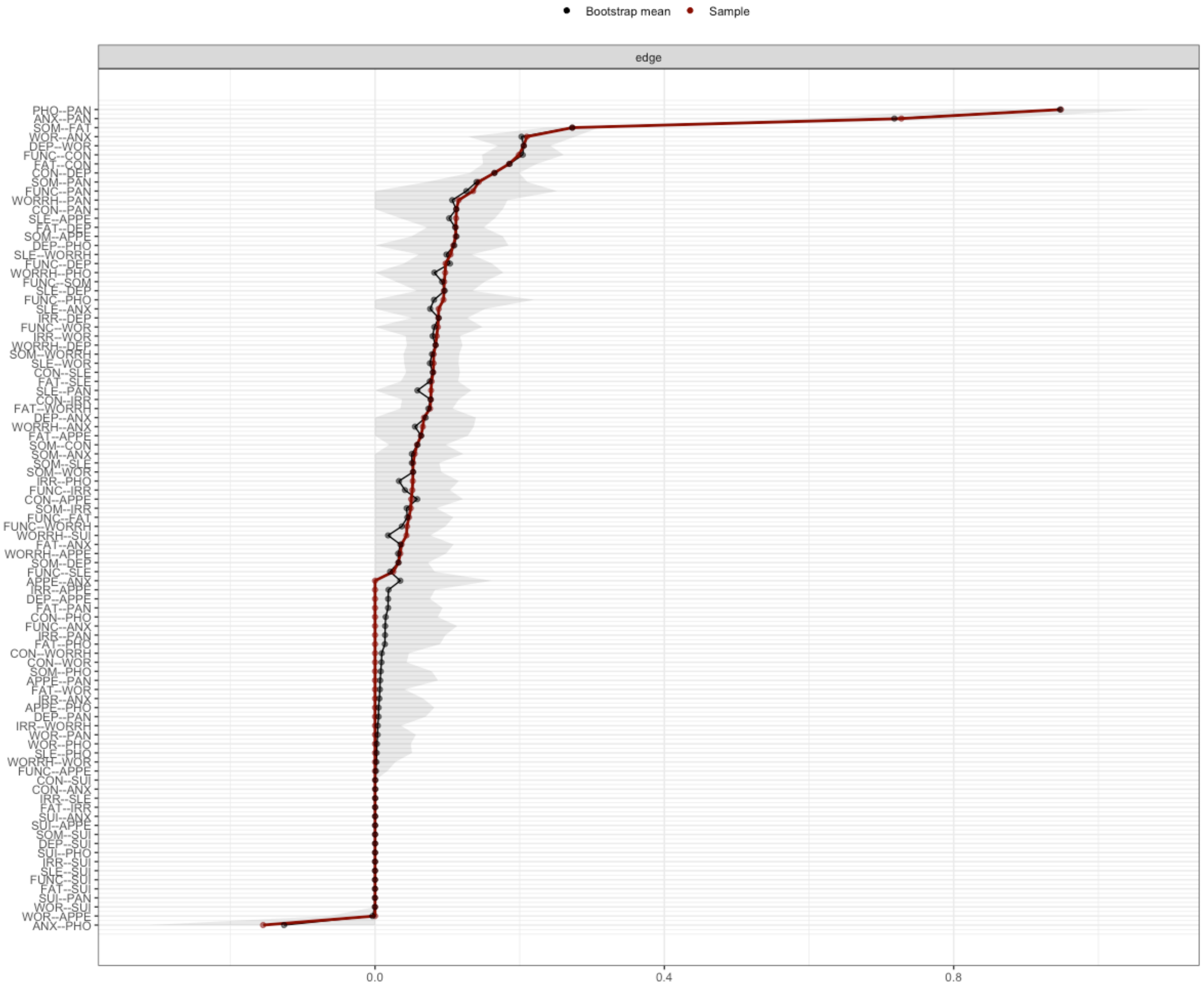

Figure 26. The MGM network, bootstrapped edge-weights, the CIs, and the percentage of times an edge was estimated as zero (edge-weight stability)

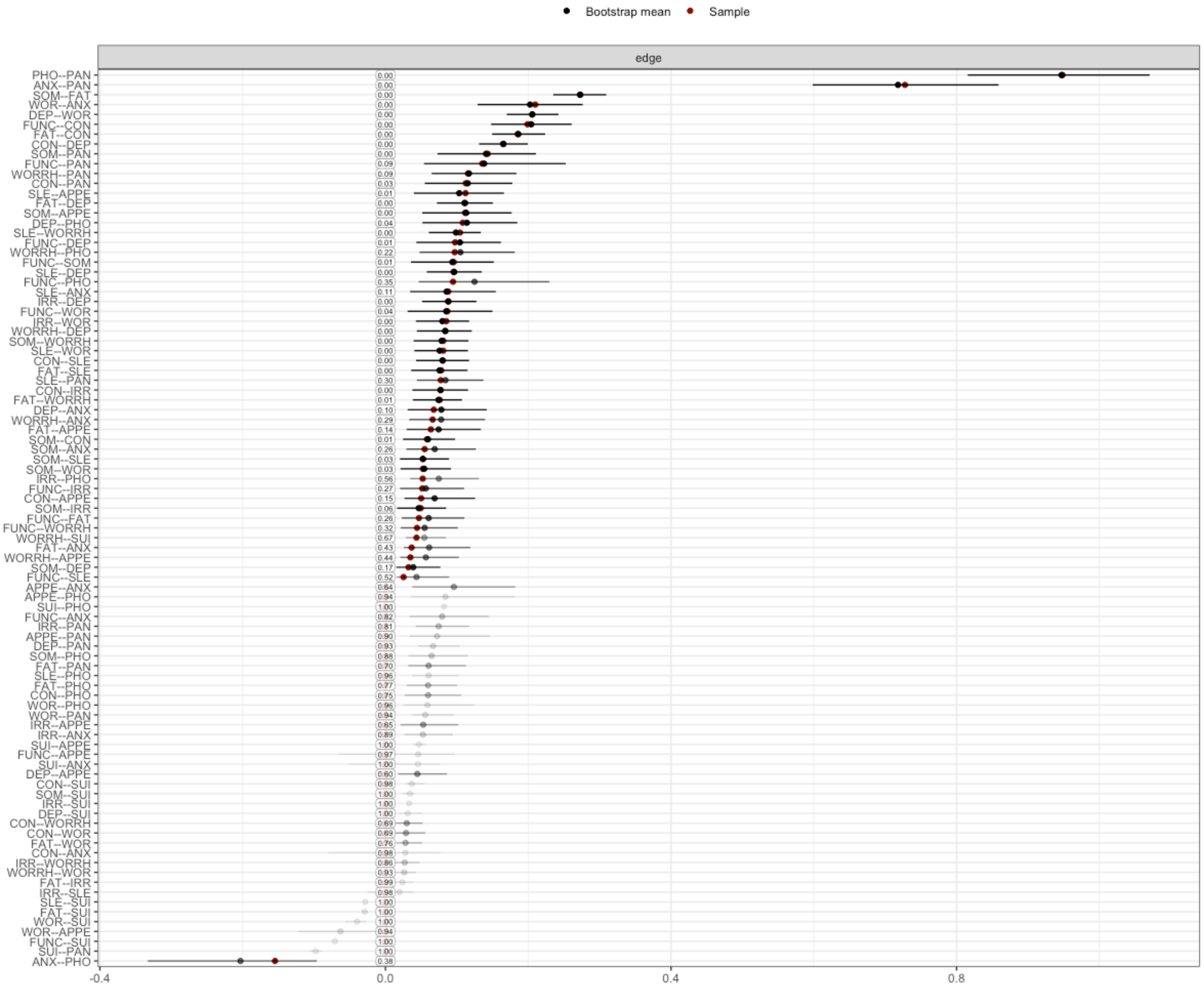

Figure 27. The MGM network, bootstrapped differences of the edge-weights (black boxes indicate significant difference)

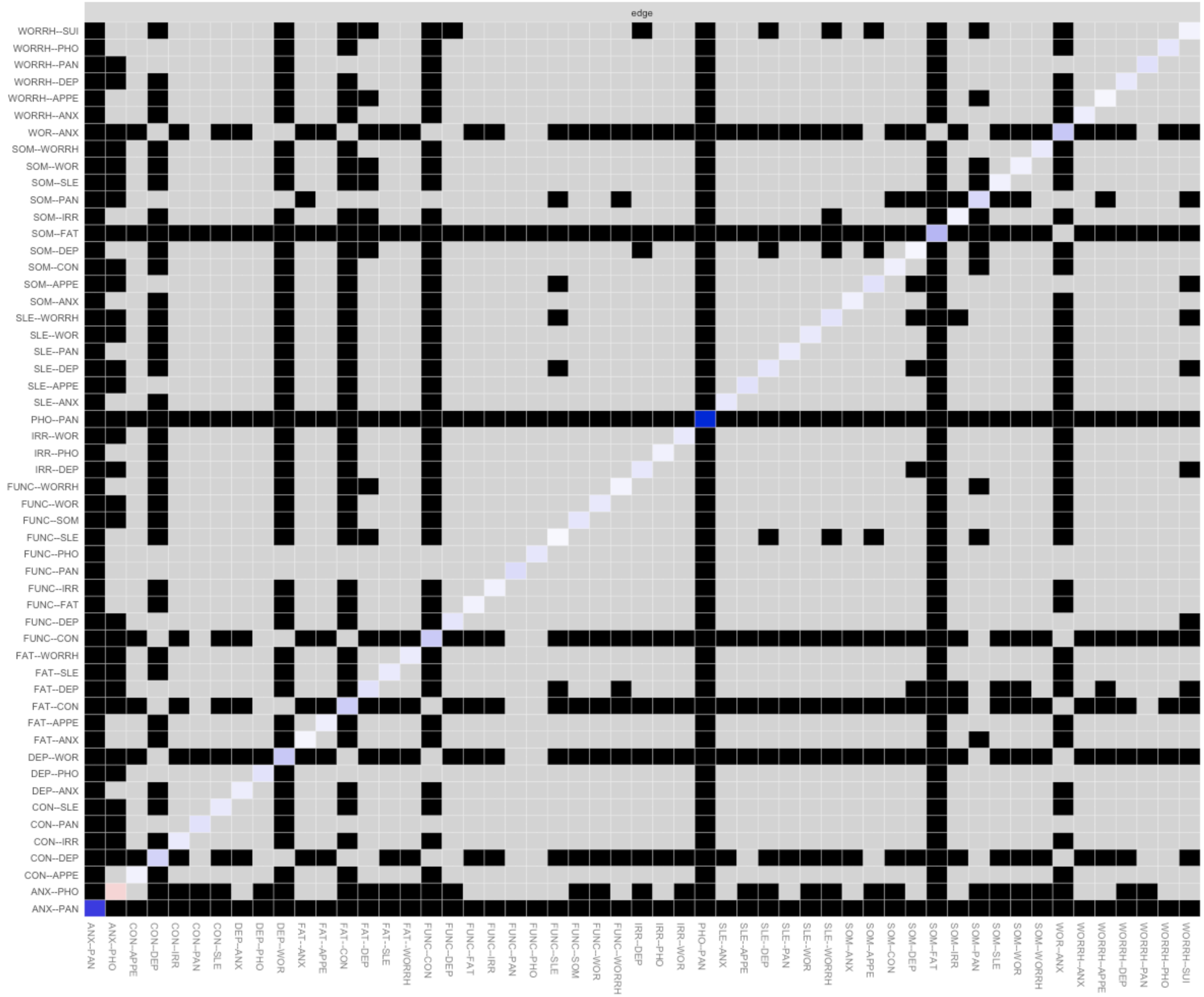

Figure 28. Centrality indices (standardized values) across three models, mgm, ggm, Ising model

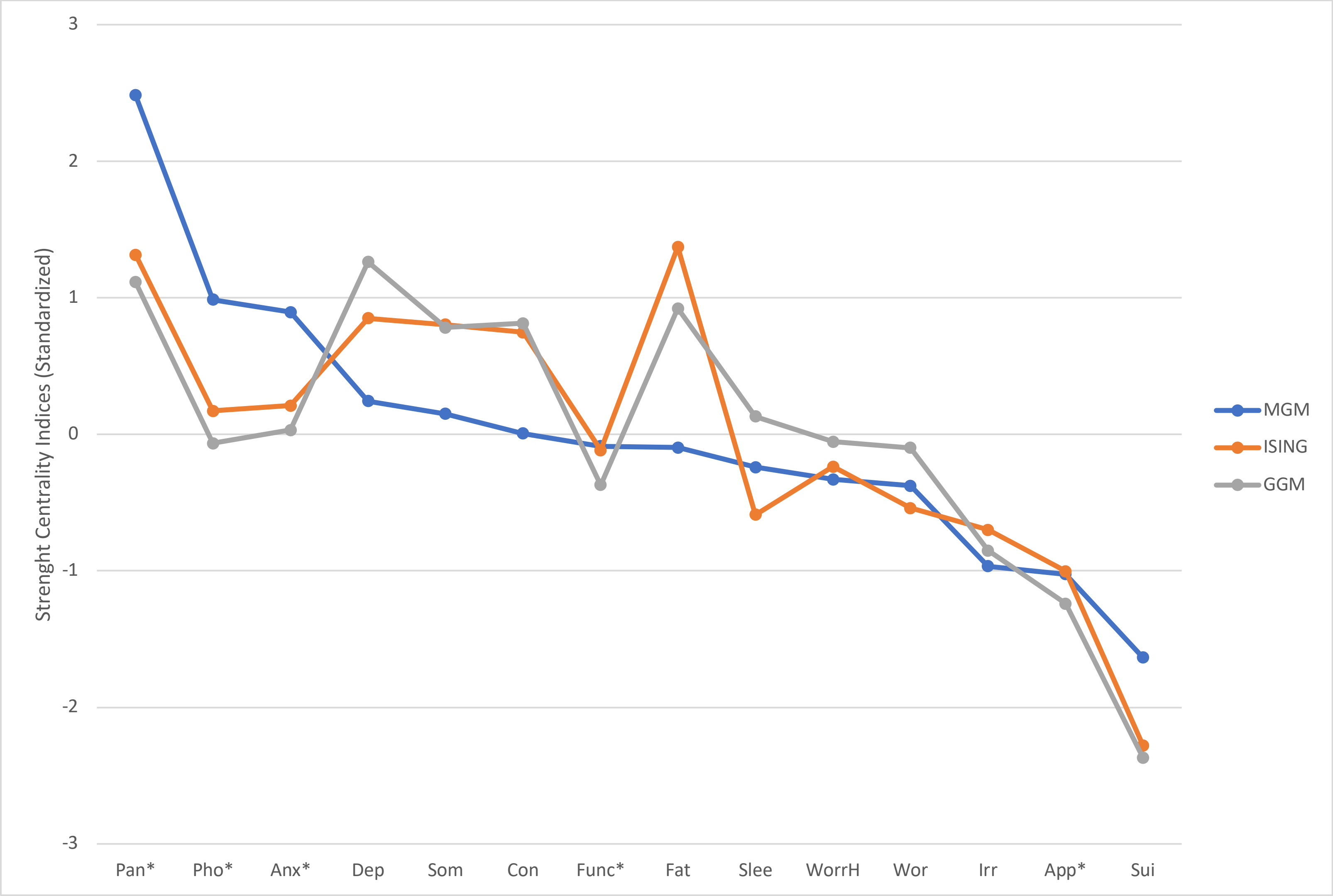

Supplement: Sönmez et al. supplementary material [file S2054425125000160sup001.pdf]
